# Cyr61 from adipose‐derived stem cells promotes colorectal cancer metastasis and vasculogenic mimicry formation *via* integrin α_V_β_5_

**DOI:** 10.1002/1878-0261.12998

**Published:** 2021-06-01

**Authors:** Zhenxing Liang, Huashan Liu, Yunfeng Zhang, Li Xiong, Ziwei Zeng, Xiaowen He, Fengwei Wang, Xianrui Wu, Ping Lan

**Affiliations:** ^1^ Department of Colorectal Surgery The Sixth Affiliated Hospital Sun Yat‐sen University Guangzhou China; ^2^ Guangdong Provincial Key Laboratory of Colorectal and Pelvic Floor Diseases The Sixth Affiliated Hospital Sun Yat‐sen University Guangzhou China; ^3^ Bioland Laboratory Guangzhou Regenerative Medicine and Health Guangdong Laboratory Guangzhou China; ^4^ Department of Endocrinology The First Affiliated Hospital of Sun Yat‐sen University Guangzhou China; ^5^ State Key Laboratory of Oncology in South China Sun Yat‐sen University Cancer Center Guangzhou China

**Keywords:** adipose‐derived stem cells, colorectal cancer, Cyr61, metastasis and vasculogenic mimicry formation

## Abstract

Adipose‐derived stem cells (ADSCs) play a vital role in colorectal cancer (CRC) progression, but the mechanism remains largely unknown. Herein, we found that ADSCs isolated from CRC patients produced more cysteine‐rich 61 (Cyr61) than those from healthy donors, and the elevated serum Cyr61 levels were associated with advanced TNM stages. Moreover, serum Cyr61 displayed a better diagnostic value for CRC compared to carcinoembryonic antigen (CEA) and carbohydrate antigen (CA19‐9). Mechanistically, integrin α_V_β_5_ was identified as the functional receptor by which Cyr61 promotes CRC cell metastasis *in vitro* and *in vivo* by activating the α_V_β_5_/FAK/NF‐κB signaling pathway. In addition, Cyr61 promotes vasculogenic mimicry (VM) formation, thereby promoting tumor growth and metastasis through a α_V_β_5_/FAK/HIF‐1α/STAT3/MMP2 signaling cascade. Histologically, xenografts and clinical samples of CRC both exhibited VM, which was correlated with HIF‐1α and MMP2 activation. Notably, we demonstrated the synergistic effect of combined anti‐VM therapy (integrin α_V_β_5_ inhibitor) and anti‐VEGF therapy (bevacizumab) in patient‐derived xenograft models. Further investigation showed that CRC cell‐derived exosomal STAT3 promoted Cyr61 transcription in ADSCs. These findings indicate that Cyr61 derived from ADSCs plays a critical role in promoting CRC progression *via* integrin α_V_β_5_ and provides a novel antitumor strategy by targeting Cyr61/α_V_β_5_.

## Graphical abstract

Cysteine‐rich 61 (Cyr61) derived from adipose‐derived stem cells (ADSCs) promotes colorectal cancer (CRC) metastasis and vasculogenic mimicry *via* binding to the integrin α_V_β_5_ receptor and activating the FAK signaling pathway. In turn, CRC cell‐derived exosomal STAT3 and p‐STAT3 promote Cyr61 transcription in ADSCs. These findings suggest that targeting of Cyr61/α_V_β_5_ may serve as a novel antitumor strategy in patients with CRC.

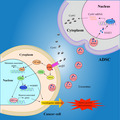

**Abbreviations**
ADSCs: adipose‐derived stem cells
AUC: area under the ROC curve
CRC: colorectal cancer
CTCs: circulating tumor cells
Cyr61: cysteine‐rich 61
PAS: periodic acid‐Schiff
PDX: patient‐derived xenograft
ROC: receiver operating characteristic
VM: vasculogenic mimicry

## 1 Introduction

Colorectal cancer (CRC) is one of the most common carcinomas worldwide, accounting for 8% of cancer incidence and cancer‐related deaths in 2018 [1]. Distant metastasis accounts for ˜ 90% of cancer‐related deaths due to limited treatment options [2, 3]. However, the molecular mechanisms of metastasis remain to be fully elucidated.

Adipose tissue is an endocrine organ, and obesity is a national and international public health concern now [4, 5]. Obesity has been reported to be a risk factor for several chronic diseases, including colon cancer [6]. Adipose‐derived stem cells (ADSCs) exist widely in adipose tissue and have the ability to differentiate into numerous cells [7]. The impacts of ADSCs on cancer progression are controversial. Although some studies have shown a protective effect of ADSCs mediated by suppressing tumor growth and stimulating apoptosis [8], most studies held the opinion that ADSCs promote tumor progression by influencing the tumor microenvironment [9, 10, 11, 12]. ADSCs can differentiate into carcinoma‐associated fibroblasts to promote tumor proliferation [13, 14]. In addition, ADSCs secrete multiple cytokines and growth factors, such as TGFβ1, insulin‐like growth factor, and VEGF, which contribute to aggressive tumor behavior [15, 16, 17]. Nevertheless, some novel ADSCs secreted cytokines still remain unexploited.

Cysteine‐rich 61 (Cyr61), also known as CCN1, belongs to the CCN protein family. Previous studies found that Cyr61 was upregulated in the serum of certain cancers and associated with poor prognosis, such as breast cancer [18], gastric cancer [19], hepatocellular carcinoma [20], and CRC [21]. Cyr61 has diverse biological functions, including promoting cell migration, proliferation, survival, and differentiation, through binding to cell‐specific integrin receptors [22]. For example, in endothelial cells and vascular smooth muscle cells, Cyr61 stimulates cell migration *via* binding to α_v_β_3_ and α_6_β_1_ [23]. In gastric adenocarcinoma cells, Cyr61 activates the NF‐κB/cyclooxygenase‐2 signaling pathway through binding to integrin α_v_β_3_ [24]. In CRC, Cyr61 has been shown to be upregulated and can cooperate with integrin α_V_β_5_ to promote CRC cell migration [25, 26, 27]. Estrada *et al*. [28] reported that Cyr61 which is secreted by bone marrow‐derived mesenchymal stem cells is able to promote angiogenesis. However, the biological functions, whether integrin α_V_β_5_ was the functional receptor, and source of Cyr61 in CRC remain not fully clarified.

In this study, we found that CRC‐associated ADSCs (ADSCs‐CRC) secreted more Cyr61 than the controls (ADSCs‐NC). Elevated serum Cyr61 levels were associated with advanced TNM stages. Mechanistically, we identified that integrin α_V_β_5_ was the functional receptor of Cyr61 and Cyr61 promotes CRC metastasis and vasculogenic mimicry (VM) formation by activing the signaling pathway downstream of integrin α_V_β_5_. Moreover, synergistic effect of anti‐VM by integrin α_V_β_5_ inhibitor and anti‐VEGF by bevacizumab therapy was found in patient‐derived xenograft (PDX) models. Collectively, our findings indicate that Cyr61 derived from ADSCs plays a critical role in promoting CRC progression *via* integrin α_V_β_5_ and provides a novel antitumor strategy based on targeting the Cyr61/α_V_β_5_.

## 2 Material and methods

### 2.1 ADSC isolation and characterization

After obtaining informed consent, adipose tissues were obtained from omentum majus from CRC patients and control donors undergoing surgery for non‐neoplastic disease and were split as previously studies [29]. First, adipose tissues were washed with PBS to remove debris and red blood cells. Adipose tissues were cut into pieces and treated with 0.25% collagenase type I for 30 min at 37 °C. Then, equal volume Dulbecco's modified Eagle medium (DMEM; Gibco, Thermo Fisher Scientific, St Peters, MO, USA) with 10% FBS (Gibco) was added to neutralize the collagenase activity. Finally, cells were plated on dishes in DMEM with 1 g·L^−1^ glucose and 10% FBS. After the third passage, we identified the cells by flow cytometric analysis with three stem cell positive markers, CD105, CD90, and CD73, and seven negative markers CD45, CD79a, CD19, CD34, CD14, CD11b, and HLA‐DR.

### 2.2 Patients and samples

Cyr61 levels were analyzed in serum samples from healthy donors (*n* = 90) and CRC patients (*n* = 364). Integrin β_5_ levels were analyzed in 10 paired CRC tissue samples. 293 samples of CRC formalin‐fixed, paraffin‐embedded tissues were used for integrin β_5_ expression detection and Kaplan–Meier survival analysis. None of the patients received chemotherapy or radiotherapy before surgery. All the samples were collected from the Sixth Affiliated Hospital of Sun Yat‐sen University. All samples were stored at −80 °C refrigerator until further use.

### 2.3 Cell culture and cell treatment

The CRC cell lines HCT8, HCT116, DLD1, and human embryonic kidney 293T cells were purchased from the American Type Culture Collection (ATCC, Washington, DC, USA). All cells were cultured in DMEM (Gibco) supplemented with 10% FBS (Gibco) at 37 °C under 5% CO_2_. Recombinant human Cyr61 (rCyr61) protein was purchased from Amyjet Scientific. The neutralizing anti‐Cyr61 antibody and the integrin β_5_ inhibitor (EMD121974; EMD) were purchased from Thermo Fisher Scientific and Selleck (Houston, TX, USA), respectively. Different concentrations of rCyr61, neutralizing antibody, and inhibitor were added prior to experiments.

### 2.4 ELISA

Cyr61 ELISA kit (RayBiotech, Atlanta, GA, USA) was used to detect the Cyr61 level in supernatants of ADSCs and serum samples according to the manufacturer's protocol. elisacalc software (ELISA Calc, Shanghai, China) was used to generate standard curve.

### 2.5 RNA extraction and real‐time PCR

Total RNA was isolated from cells by TRIzol Reagent (Thermo Fisher Scientific). ReverTra Ace qPCR RT Kit (Toyobo, Kita‐ku, Osaka, Japan) was used to perform reverse transcription according to the manufacturer's instructions. The Applied Biosystems 7500 Sequence Detection system was used to carry out quantitative real‐time reverse transcription PCR (qRT‐PCR) with the SYBR Green PCR Master Mix (Applied Biosystems, Foster City, CA, USA). We generated standard curves and applied the 2^−▵▵CT^ method with normalized to 18S rRNA. All the gene‐specific primers were obtained from Invitrogen (Thermo Fisher Scientific), and the oligonucleotide sequences are listed in Table S4.

### 2.6 Western blot analysis

Cell and tissue samples were lysed with radio‐immunoprecipitation assay buffer (RIPA) with protease and phosphatase inhibitor cocktail (Promega, Fitchburg, WI, USA). Proteins were separated by SDS/PAGE and then transferred to polyvinylidene fluoride (PVDF) membranes by the Trans‐Blot System (Bio‐Rad, Hercules, CA, USA). The membranes were blocked by milk and then incubated with specific primary antibodies against Cyr61 (Abcam, Cambridge, MA, USA, 1 : 1000), integrin β_5_ (Cell Signaling Technology, Danvers, MA, USA, CST, 1 : 1000), FAK (CST, 1 : 1000), p‐FAK (CST, 1 : 1000), P65 (CST, 1 : 1000), GAPDH (Abcam, 1 : 1000), MEK (CST, 1 : 1000), p‐MEK (CST, 1 : 1000), ERK (CST, 1 : 1000), p‐ERK (CST, 1 : 1000), STAT3 (CST, 1 : 1000), p‐STAT3 (CST, 1 : 1000), MMP2 (Abcam, 1 : 2000), and HIF‐1α (Abcam, 1 : 500). Finally, membranes were incubated with a specific secondary antibody and visualized by ECL Blotting Detection Reagents. GAPDH served as a control for western blot analysis.

### 2.7 Cell linages isolation in CRC tissues

Fresh tissue was chopped with a sterile scalpel and then digested for 1 h at 37 °C using collagenase digestion medium (RPMI‐medium, collagenase type IV 1 mg·mL^−1^ and DNAse I 150 U·mL^−1^) to obtain single cell suspension. Lymphocytes, macrophages, endothelial cells, and CRC cells were purified with CD3^+^ microbeads (Miltenyi Biotec, Bergisch Gladbach, Germany, 130‐050‐101), CD14^+^ microbeads (Miltenyi Biotec, 130‐050‐201), CD31^+^ microbeads (Miltenyi Biotec, 130‐091‐935), and EpCAM^+^ microbeads (Miltenyi Biotec, 130‐061‐101), respectively. Finally, cells were plated in adherent conditions in growth medium (DMEM, Pen‐Strep 1X, 10% FBS) and passaged regularly to obtain fibroblasts.

### 2.8 Cell migration assay

Cell migration assays were performed with 24‐well plates with 8 μm pore size chamber inserts (Corning, New York, NY, USA). In general, 5 × 10^4^ cells resuspended with 200 μL serum‐free DMEM were seeded in the upper chamber well and 800 μL of DMEM with 10% FBS was added into the lower chamber. After 24 h, cells migrating through the membrane were fixed with 4% paraformaldehyde for 15 min and then stained with 0.1% crystal violet for 15 min. The cells were viewed under an inverted microscope (DMI4000B; Leica, Wetzlar, Germany) and quantified using software imagej (Bethesda Softworks LLC, Rockville, MD, USA).

### 2.9 Wound‐healing assay

A total of 2 × 10^6^ cells were seeded in six‐well plates and incubated until confluency was reached. A 100 μL pipette tip was used to create a rectilinear scratch. After 24 h, cells were fixed with 4% paraformaldehyde for 15 min and then stained with 0.1% crystal violet for 15 min. An inverted microscope (DMI4000B; Leica) was utilized to image the wound closure.

### 2.10 Cell counting

A total of 5 × 10^4^ cells were resuspended in DMEM with 10% FBS and seeded in a 12‐well plate. The cells were released by trypsinization 3 days later, and cell numbers were counted immediately.

### 2.11 Vector construction and generation of stable cell lines

The methods for vector construction and generation of stable cell lines were described in our previous study [30]. The oligonucleotides to suppress integrin β_5_ expression were designed by RiboBio (Guangzhou, China). After we verified their knockdown efficiency, they were cloned into lentiviral expression vector pLKO.1‐Pur (Addgene, Cambridge, MA, USA). The plasmids were verified by sequencing. Empty vector pLKO.1‐Pur carrying a scrambled shRNA served as a control. 293T cells were incubated with the constructed vectors, pMD2G and psPAX2 (Addgene), according to the manufacturer's protocol. 0.22 μm PVDF filters were used to filter 293T cells supernatant, and then, the supernatant was added into the plate to infect DLD1, HCT116, and HCT8 cells. The oligonucleotide sequences for vector construction are listed in Table S4.

### 2.12 Mass spectrometry assay

The gel was chopped into small fragments with a razor blade, destained, and subjected to digestion by modified porcine trypsin (50–100 ng per digestion; Promega). After trypsin digestion, peptides were dissolved in 0.1% FA and 2% ACN, directly loaded onto a reversed‐phase analytical column (75 μm i.d. × 150 mm, packed with Acclaim PepMap RSLC C18, 2 μm, 100 Å, nanoViper, Thermo Fisher Scientific). The gradient was comprised of an increase from 5% to 50% solvent B (0.1% FA in 80% ACN) over 40 min, climbing to 90% in 5 min, and then holding at 90% for the 5 min. All at a constant flow rate of 300 nL·min^−1^. The MS analysis was performed on Q Exactive hybrid quadrupole‐Orbitrap mass spectrometer (Thermo Fisher Scientific). The peptides were subjected to NSI source followed by tandem mass spectrometry (MS/MS) in Q ExactiveTM (Thermo Fisher Scientific) coupled online to the UPLC. Intact peptides were detected in the Orbitrap at a resolution of 70 000. Peptides were selected for MS/MS using NCE setting as 27; ion fragments were detected in the Orbitrap at a resolution of 17 500. A data‐dependent procedure that alternated between one MS scan followed by 20 MS/MS scans was applied for the top 20 precursor ions above a threshold ion count of 1E4 in the MS survey scan with 30.0s dynamic exclusion. The electrospray voltage applied was 2.0 kV. Automatic gain control (AGC) was used to prevent overfilling of the ion trap; 1E5 ions were accumulated for generation of MS/MS spectra. For MS scans, the *m*/*z* scan range was 350–1800 *m*/*z*. Fixed first mass was set as 100 *m*/*z*. Protein identification was performed with MASCOT software by searching Uniprot_Aedis Aegypti.

### 2.13 Membrane protein extraction and receptor identification

Cell membrane proteins were extracted using the ProteoExtract Native Membrane Protein Extraction Kit (M‐PEK Kit; Calbiochem, Thermo Fisher Scientific) according to manufacturer’s recommendation. Before this experiment, cells were treated with 40 ng·mL^−1^ rCyr61 for 1 h. The IgG group and 0.1% SDS group served as controls. The SDS‐Out Precipitation Kit (Pierce, Thermo Fisher Scientific) was used to remove the SDS. Then, the extracts were incubated with 50ul beads coupled with IgG or anti‐human monoclonal Cyr61 antibody at 37 °C overnight. Finally, proteins were separated by SDS/PAGE and visualized by Coomassie blue staining. The protein‐specific bands were excised for mass spectrometric analysis.

### 2.14 Immunohistochemistry and IHC scoring

Paraffin‐embedded tissues were deparaffinized with dimethylbenzene followed by antigen retrieval. The tissues were blocked with normal goat serum at 37 °C for 30 min. Next, the tissues were incubated overnight at 4 °C with specific primary antibodies against integrin β_5_ (Bioss, Boston, MA, USA, 1 : 200), p‐FAK (CST, 1 : 600), p‐ERK (CST, 1 : 250), p‐STAT3 (CST, 1 : 200), MMP2 (Abcam, 1 : 200), HIF‐1α (Abcam, 1 : 100), and CD31 (CST, 1 : 200). Finally, the tissues were incubated with appropriate secondary antibodies and then incubated with 3, 3′‐diaminobenzidine (DAB). We evaluated the marker staining results according to a previous study [31]. The staining intensity was graded 4 stages: 0 (none), 1 (weak), 2 (moderate), and 3 (strong). The percentage of expression was graded 5 stages: 0 (< 5% staining), 1 (5–25% staining), 2 (25–50% staining), 3 (50–75% staining), and 4 (> 75% staining). The sum of both scores served as the final score. Two pathologists performed the scoring analyses according to the above criteria.

### 2.15 Immunofluorescence assays

The cells were incubated overnight at 4 °C with specific primary antibodies Cyr61 (Abcam, 1 : 500), integrin β5 (CST, 1 : 1600), p‐FAK (CST, 1 : 100), P65 (CST, 1 : 400), p‐MEK (CST, 1 : 500), ERK (CST, 1 : 800), STAT3 (CST, 1 : 100), MMP2 (Abcam, 1 : 250), and HIF‐1α (Abcam, 1 : 1000). Then, cells were incubated with Alexa 488‐ or Alexa 594‐conjugated goat antibodies (Thermo Fisher Scientific) against mouse or rabbit IgG. Finally, the samples were counterstained with DAPI and imaged with confocal laser‐scanning microscope (Leica TCS‐SP8).

### 2.16 *In vitro* vasculogenic mimicry assay

Briefly, 30 μL of Matrigel (BD, BD Biosciences, Franklin Lakes, NJ, USA) was plated in 96‐well plates and incubated at 37 °C for 30 min to allow polymerization. Next, 1 × 10^5^ cells per well were added to the Matrigel layer and grown for 12 h. Randomized fields were captured using an inverted microscope, and tubes were quantified from each image.

### 2.17 Flow cytometric analysis

Adipose‐derived stem cells were incubated with fluorochrome‐conjugated specific antibodies and matched control IgG at room temperature for 30 min. Then, flow cytometry (BD Biosciences) was used to analyze the cells. The data were analyzed by software flowjo software (v10.0.7, Tree Star, San Carlos, CA, USA).

### 2.18 Luciferase assays

Cyr61 promoter sequences (from −2000 to +100 relative to the transcription start site) and sequential deletion were cloned into pGL3‐Basic vector. STAT3 cDNA was cloned in pcDNA3.1. These plasmids were transfected into ADSCs by Lipofectamie 3000 (Invitrogen) according to the manufacturer's instructions. A Dual‐luciferase Reporter Assay System (Promega) was used according to the manufacturer's instructions.

### 2.19 Exosome extraction and identification

Exosomes were extracted and identified according to our previous study [30]. In briefly, supernatants were harvested and centrifuged sequentially at 300 ***g*** for 15 min, 2000 ***g*** for 15 min, 10 000 ***g*** for 30 min, and 120 000 ***g*** for 70 min (Beckman Coulter, Brea, CA, USA) twice. Then, Particle Metrix (PMX), transmission electron microscopy (TEM), and western blot analysis were used to identify the exosomes.

### 2.20 Animal experiments

All animal experiments were approved by the Institutional Animal Care and Use Committee of Sun Yat‐sen University, Guangzhou, China. Four‐ to five‐week‐old male BALB/c nude mice were purchased from the Animal Experiment Center of Sun Yat‐Sen University and were fed in SPF environment. A spleen injection model was used for liver colonization assays (*n* = 5 per group). We analyzed liver metastasis by autopsy and hematoxylin and eosin staining (H&E). After CRC cells were treated with Cyr61 (20 ng·mL^−1^) for one week, 1 × 10^6^ cells in 100 μL PBS were intrasplenically injected. An immediate splenectomy was performed following intrasplenic injection. Subcutaneous tumor growth assays were performed as previous study [32] (*n* = 5 per group). Subcutaneous tumors were evaluated by H&E and immunohistochemistry (IHC). GFP‐positive circulating tumor cells (CTCs) in PBMCs of mice were analyzed by flow cytometry as the previous study [33].

### 2.21 Patient‐derived xenograft models and intratumoral injection assay

Fresh tumor tissues were obtained from two CRC patients and implanted into NCG mice. When the tumor size reached 1.5 cm^3^, the tumors were divided into equal volume ˜ 2 mm^3^ and were subcutaneously implanted into 4‐ to 5‐week‐old male BALB/c nude mice. When the tumor size reached about 100 mm^3^, all mice were randomized into four groups (*n* = 4 per group): control group, cilengitide (EMD121974, 10 mg·kg^−1^, once a week) group, bevacizumab (5 mg·kg^−1^, once a week), and EMD121974 plus bevacizumab group. Intratumor injection of rCyr61 (0.2 μg·kg^−1^) was performed 24h later after inhibitor intratumor injection. All reagents were injected precisely into the center of the tumors. All mice were sacrificed 4 weeks later, and subcutaneous tumors were subjected to H&E and IHC.

### 2.22 Statistical analysis

graphpad prism Software (GraphPad Software, La Jolla, CA, USA) was used to perform statistical analysis. Two‐tailed Student's test, one‐way ANOVA, and Pearson's correlation analysis were performed for statistical comparisons. All statistics analysis data are expressed as mean ± standard error of the mean. A *P* value < 0.05 was considered statistically significant.

### 2.23 Study approval

All samples from human tissues were collected with written informed consent from donors, and all procedures were performed with the approval of the Institutional Review Board of The Sixth Affiliated Hospital of Sun Yat‐sen University. Animal experiments were approved by the Institutional Animal Care and Use Committee of Sun Yat‐sen University and conformed to the Guide for the Care and Use of Laboratory Animals of the National Institutes of Health (National Academies Press, 2011) in China.

## 3 Results

### 3.1 Cyr61 expression is upregulated in ADSCs derived from CRC patients

We first identified the characteristics of the ADSCs after isolation. ADSCs exhibited a typical spindle‐shaped fibroblast‐like appearance (Fig. S1A). Furthermore, ADSCs possessed the capacity for multilineage differentiation into adipocytes (Fig. S1B) and osteocytes (Fig. S1C). Flow cytometric analysis of the cell surface markers showed that the isolated ADSCs were positive for CD105, CD90, and CD73 and were negative for CD45, CD79a, CD19, CD34, CD14, CD11b, and HLA‐DR (Fig. S1D). Thus, these data confirmed that ADSCs were successfully isolated.

**Fig. 1:**
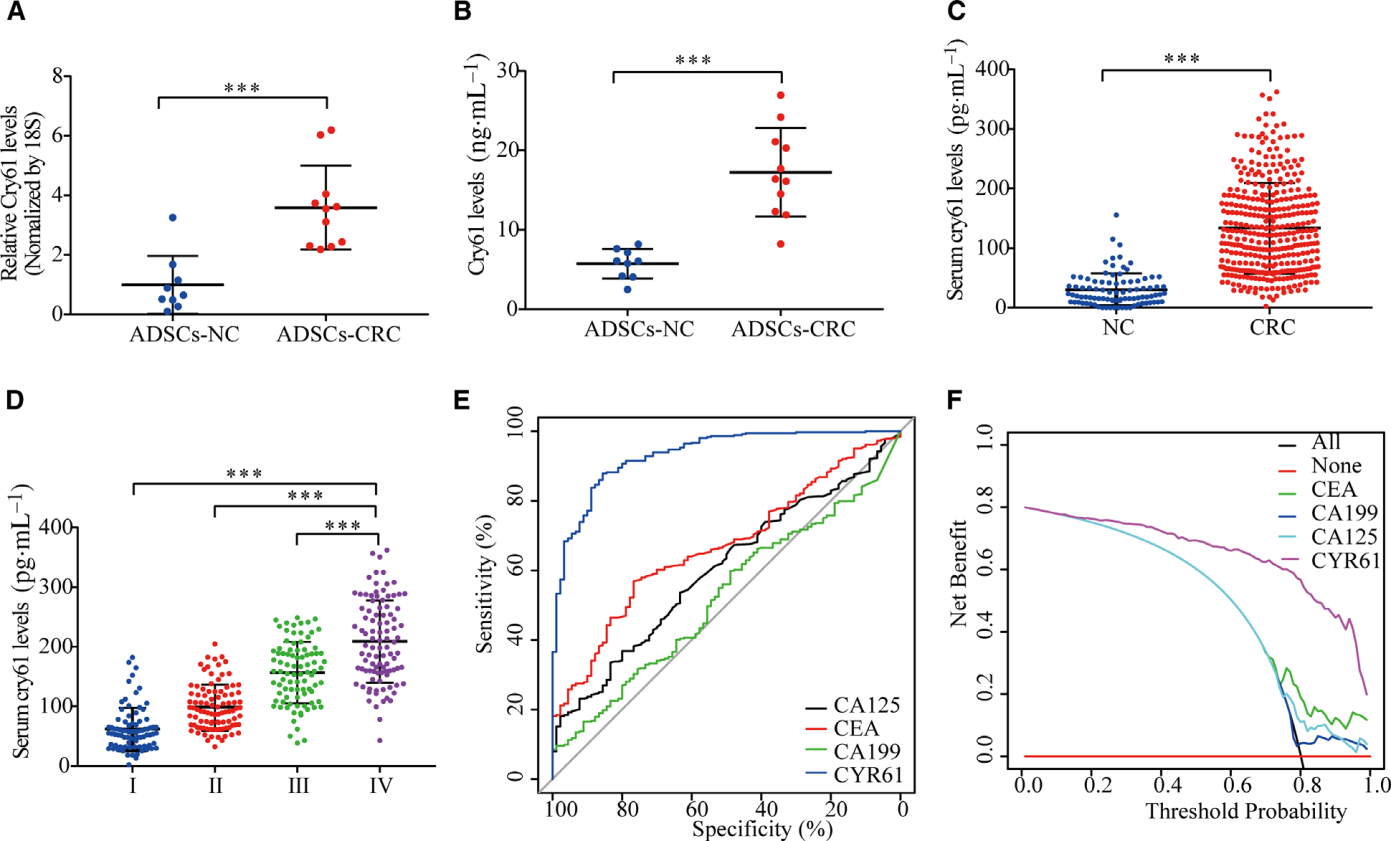
Serum Cyr61 is a diagnostic marker for CRC. (A) qRT‐PCR analysis of Cyr61 mRNA levels in ADSCs‐NC (*n* = 9) and ADSCs‐CRC (*n* = 11). (B) ELISA of Cyr61 levels in the medium of ADSCs‐NC (*n* = 9) and ADSCs‐CRC (*n* = 11). (C) ELISA of serum Cyr61 levels in healthy donors (*n* = 90) and CRC patients (*n* = 364). (D) ELISA of serum Cyr61 levels in different CRC tumor stages. (E) ROC curves for diagnosis of CRC *via* serum Cyr61, CA125, CEA, or CA199 levels. (F) Decision curve for diagnosis of CRC *via* serum Cyr61, CA125, CEA, or CA199 levels. Values are represented as mean ± SD. ***P < 0.001, by two‐tailed Student's *t*‐test (A–C) and one‐way ANOVA (D).

The CCN protein family comprises six members, including Cyr61 (CCN1), connective tissue growth factor (CTGF, CCN2), nephroblastoma overexpressed protein (Nov, CCN3), wnt‐1‐induced secreted protein 1 (WISP‐1, CCN4), WISP‐2 (CCN5), and WISP‐3 (CCN6) [34]. These proteins are involved in multiple biological processes, such as cell proliferation, migration, survival, and angiogenesis [35, 36], but the functions of these proteins in ADSCs and CRC remain to be fully clarified. We performed qRT‐PCR to analyze the mRNA levels of CCN protein family members in ADSCs isolated from nine healthy donors and 11 CRC patients. Among all the CCN protein family members tested, only Cyr61 was upregulated in ADSC‐CRC (Fig. S1E, Fig. 1A). Furthermore, ELISAs indicated that ADSCs‐CRC secreted abundant amounts of Cyr61 protein into their culture supernatants (Fig. 1B).

Next, ELISAs were performed to analyze Cyr61 protein levels in serum from 90 healthy donors and 364 CRC patients. Serum Cyr61 protein was upregulated in CRC patients and was positively correlated with tumor TNM stages (Fig. 1C,D). By assessing the relationship between serum Cyr61 and the clinicopathologic characteristics of CRC patients, we found that Cyr61 was significantly elevated in patients with more advanced TNM stages (*P* < 0.001; Table S1). We then conducted receiver operating characteristic (ROC) curve analysis to compare the diagnostic power of Cyr61 with the traditional biomarkers of CRC. The results showed that the area under the ROC curve (AUC) for Cyr61 was 0.933, better than the traditional diagnostic biomarkers (Fig. 1E and Table S2). Furthermore, decision curve analysis showed that Cyr61 levels provided greater net diagnostic power than three traditional biomarkers, regardless of the threshold used (Fig. 1F). Compared with the Cyr61 levels in ADSC culture supernatants, Cyr61 protein secreted by CRC cell lines was negligible (Fig. S1F). Moreover, western blot analysis of tumor tissues and major tumor infiltration cells, such as lymphocytes, macrophages, endothelial cells, and fibroblasts, also showed negligible expression of Cyr61 protein tissues in CRC tissues (Fig. S1G). These data demonstrated that ADSCs‐CRC appeared to be the main source of Cyr61 protein and serum Cyr61 may be a potential diagnostic biomarker of CRC.

### 3.2 ADSC‐derived Cyr61 promotes CRC cell invasion and migration *in vitro*

**Fig. 2:**
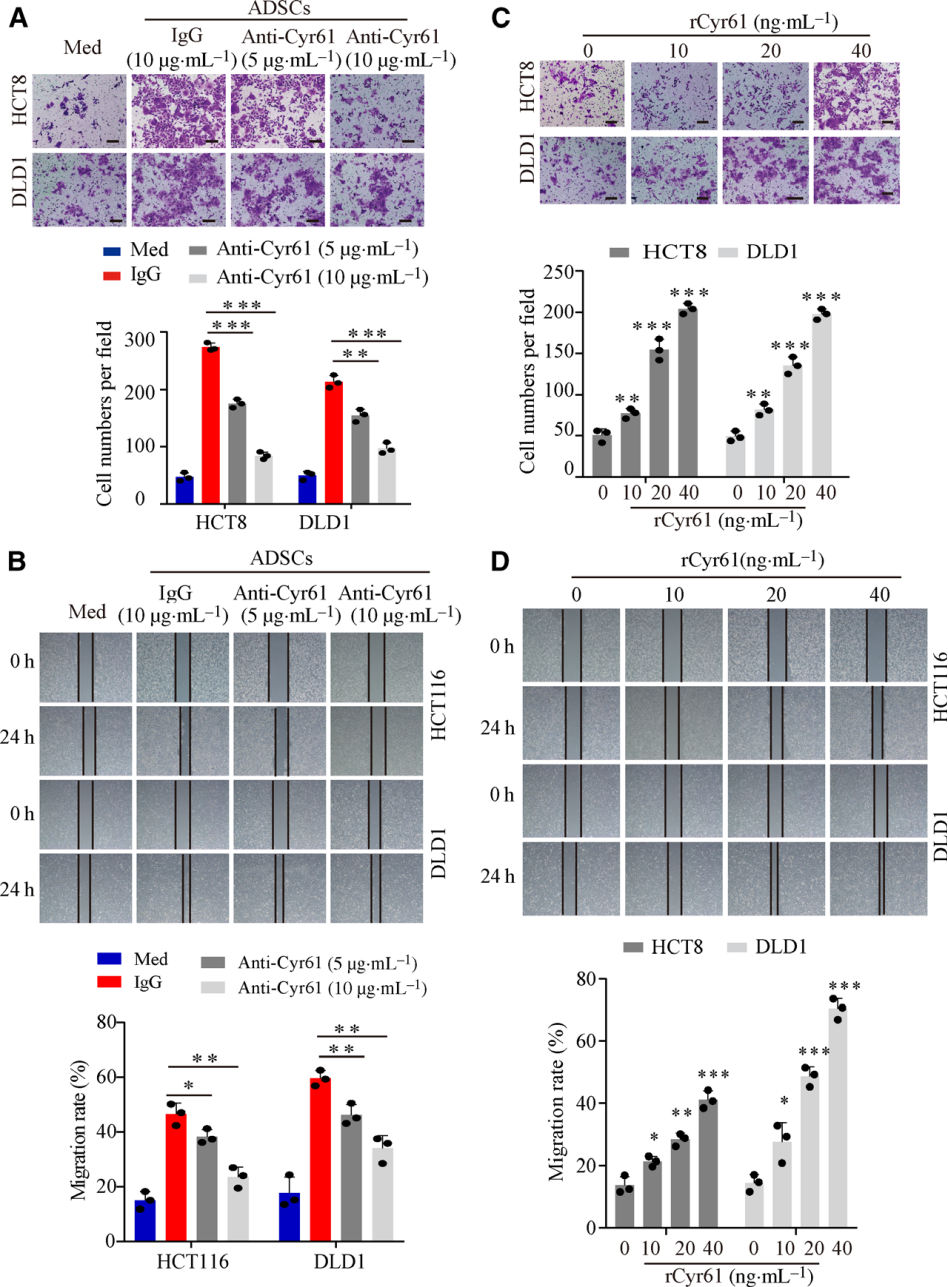
ADSC‐derived Cyr61 promotes CRC cell invasion and migration *in vitro*. (A) Representative images of transwell migration assays for HCT8 and DLD1 cells cocultured with culture medium alone (Med) or ADSCs in the presence or absence of 5 or 10 μg·mL^−1^ anti‐Cyr61 antibody, or isotype‐matched IgG control (IgG). Scale bar = 100 μm, *n* = 3. (B) Representative images of wound‐healing assays for HCT116 and DLD1 cells cocultured with Med or ADSCs in the presence or absence of an anti‐Cyr61 antibody at 5 or 10 μg·mL^−1^, or an IgG. *n* = 3 (C) Representative images of transwell migration assays for HCT8 and DLD1 cells with rCyr61 at different concentration. Scale bar = 100 μm, *n* = 3. (D) Representative images of wound‐healing assays for HCT116 and DLD1 cells with rCyr61 at different concentration. *n* = 3. Values are represented as mean ± SD. *P < 0.05, **P < 0.01, ***P < 0.001, by one‐way ANOVA.

To determine whether ADSCs contribute to cancer cell invasion and migration, we employed cell migration and wound‐healing assays. For cell migration assays, CRC cells were seeded in the upper wells with ADSCs in the lower wells. For wound‐healing assays, ADSCs were seeded in the upper wells with CRC cells in the lower wells. Compared with the ADSCs‐NC, ADSCs‐CRC significantly promoted cell invasion and migration (Fig. S2A,B). A polyclonal anti‐Cyr61 antibody with neutralized function was used to determine the ability of Cyr61 to contribute to cancer cell invasion and migration. Compared with 10 μg·mL^−1^ IgG, the addition of 5 μg·mL^−1^ anti‐Cyr61 antibody to HCT8 and DLD1 cells reduced the number of invasive cancer cells by 36% and 27%, respectively, and the addition of 10 μg·mL^−1^ anti‐Cyr61 antibody reduced the cell number by 69% and 54%, respectively (Fig. 2A). CRC cells migrated slowly to close the scratched wounds while adding neutralized anti‐Cyr61 antibody (Fig. 2B). Furthermore, treatment of HCT8, DLD1, and HCT116 cells with recombinant Cyr61 (rCyr61, 0–40 ng·mL^−1^) enhanced the invasion and migration of cells in a dose‐dependent manner (Fig. 2C,D). Cell counting and MTS assays revealed that incubation of CRC cells with ADSCs‐CRC significantly promoted cell proliferation compared to ADSCs‐CRC (Fig. S2C,D). However, adding neutralized antibody to the coculture system did not influence CRC cell proliferation (Fig. S2E,F). Collectively, these data suggested that ADSCs‐CRC promoted CRC cell invasion and migration *via* Cyr61.

### 3.3 Integrin αVβ5 is the functional receptor of Cyr61

**Fig. 3:**
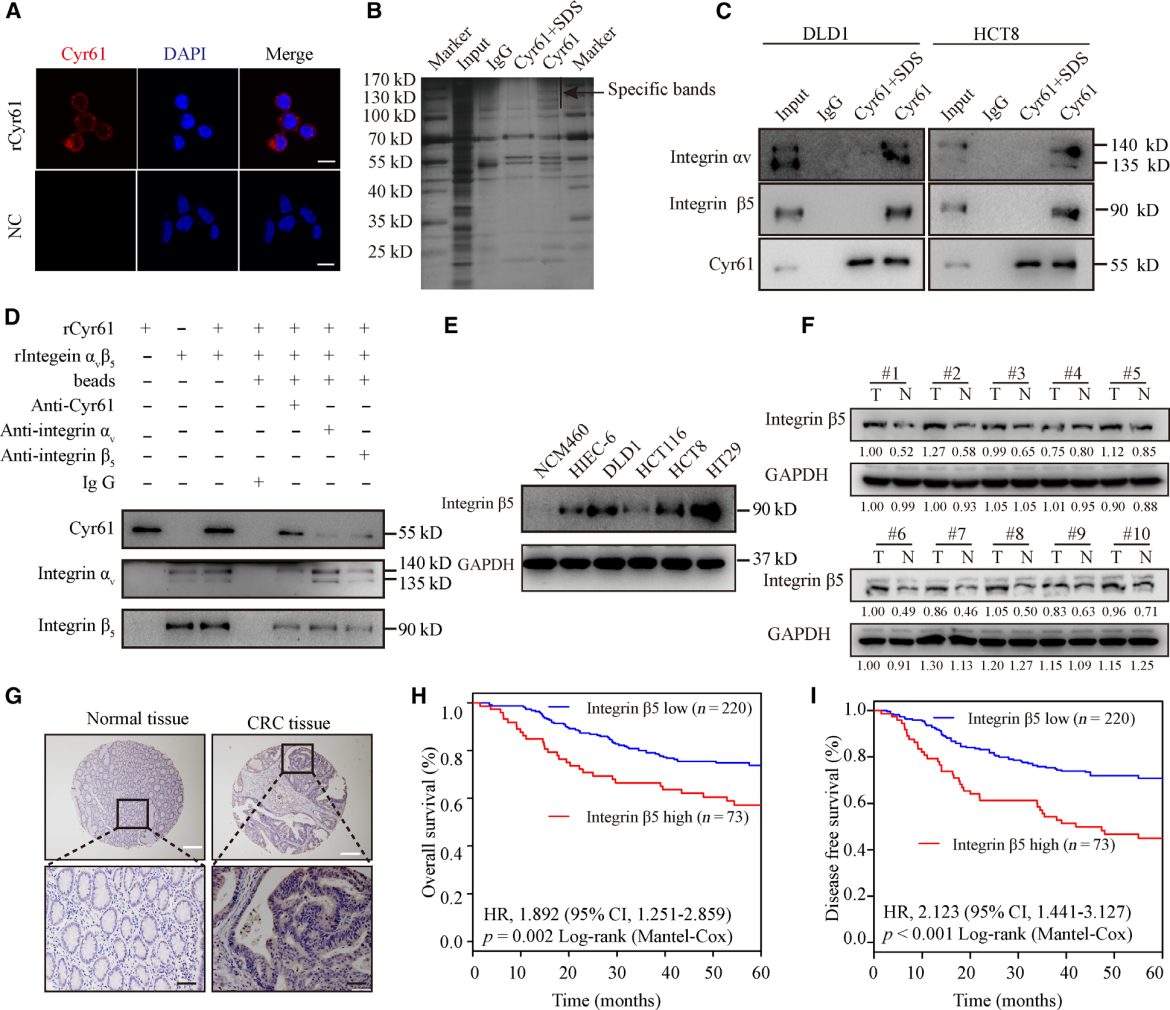
Cyr61 binds to integrin α_V_β_5_ on CRC cells. (A) Confocal microscopy for HCT8 cells in the presence or absence of rCyr61 at 40 ng·mL^−1^. Scale bar = 10 μm. (B) Immunoprecipitation of the membrane extracts of rCyr61‐treated HCT8 cells with anti‐Cyr61 antibody. (C) Western blot validation of mass spectrometric results with anti‐integrin α_V_, anti‐integrin β_5_, and anti‐Cyr61 antibody in immunoprecipitation products of the membrane extracts from rCyr61‐treated HCT8 and DLD1 cells with anti‐Cyr61 antibody. (D) Western blot analysis validation of the direct interaction between Cyr61 and integrin α_V_β_5_. (E) Western blot analysis of integrin β_5_ expression in normal colonic cell lines and CRC cell lines. (F) Western blot analysis of integrin β_5_ expression in 10 CRC tissues and paired normal adjacent tissues. (G) IHC analysis of integrin β_5_ expression in the paraffin‐embedded CRC tissues and paired normal adjacent tissues. White scale bar = 200 μm. Black scale bar = 50 μm. (H, I) Kaplan–Meier analysis for OS and DFS of CRC patients with low or high expression of integrin β_5_.

To investigate the existence of a receptor of Cyr61 on the CRC cell membrane, HCT8 cells were treated with rCyr61. Immunofluorescence (IF) assays showed that rCyr61 was localized to the cell membrane, implying the presence of a Cyr61‐specific receptor on CRC cell membrane (Fig. 3A). To identify the unknown receptor, we extracted the membranous proteins from HCT8 cells treated with rCyr61. Specific protein bands were detected after immunoprecipitation with Cyr61‐specific antibody (Fig. 3B) and were subjected to mass spectrometry. Nine candidate membranous proteins were identified (Table S3). Among these proteins, integrin α_V_β_5_ got the highest score in the mass spectrometry results and only integrin α_V_β_5_ belonged to the integrin family (Fig. S3A,B). Subsequent western blot analysis with specific antibodies for the detection of integrin α_V_ and integrin β_5_ confirmed the combination of Cyr61 with integrin α_V_β_5_ in HCT8 and DLD1 cells (Fig. 3C). Besides, purified integrin α_V_β_5_ was used to confirm the direct interaction with Cyr61 (Fig. 3D). Furthermore, CRC cells were incubated with rCyr61 and the IF assays results demonstrated that Cyr61 colocalized with integrin α_V_β_5_ on the plasma membrane (Fig. S3D). Following short hairpin RNA (shRNA)‐mediated knockdown of integrin β_5_ expression, Cyr61 localization on the cell surface was decreased (Fig. S3C,D). Moreover, integrin β_5_ was overexpressed in the majority of CRC cell lines (HCT8, DLD1, and HT29) compared to the normal colonic cell lines HIEC‐6 and NCM460 (Fig. 3E).

Next, integrin β_5_ protein levels were analyzed in 10 CRC patient tissues and matched normal tissues. Western blot analysis indicated that integrin β_5_ was notably increased in 9 out of 10 CRC tissues (Fig. 3F). To investigate the clinical significance of integrin β_5_, we detected integrin β_5_ expression levels in a large cohort of CRC patients. Immunohistochemical (IHC) analysis also suggested that integrin β_5_ was upregulated in CRC tissues (Fig. 3G). Moreover, survival analysis showed that high integrin β_5_ expression was associated with poor overall survival (OS; *P* = 0.002, Fig. 3H) and disease‐free survival (DFS) in CRC patients (*P* < 0.001; Fig. 3I). Taken together, integrin α_V_β_5_ was the functional receptor of Cyr61 and played an important role in CRC progression.

### 3.4 Cyr61 promotes CRC cell migration and invasion *via* αVβ5/FAK/NF‐κB signaling pathway

**Fig. 4:**
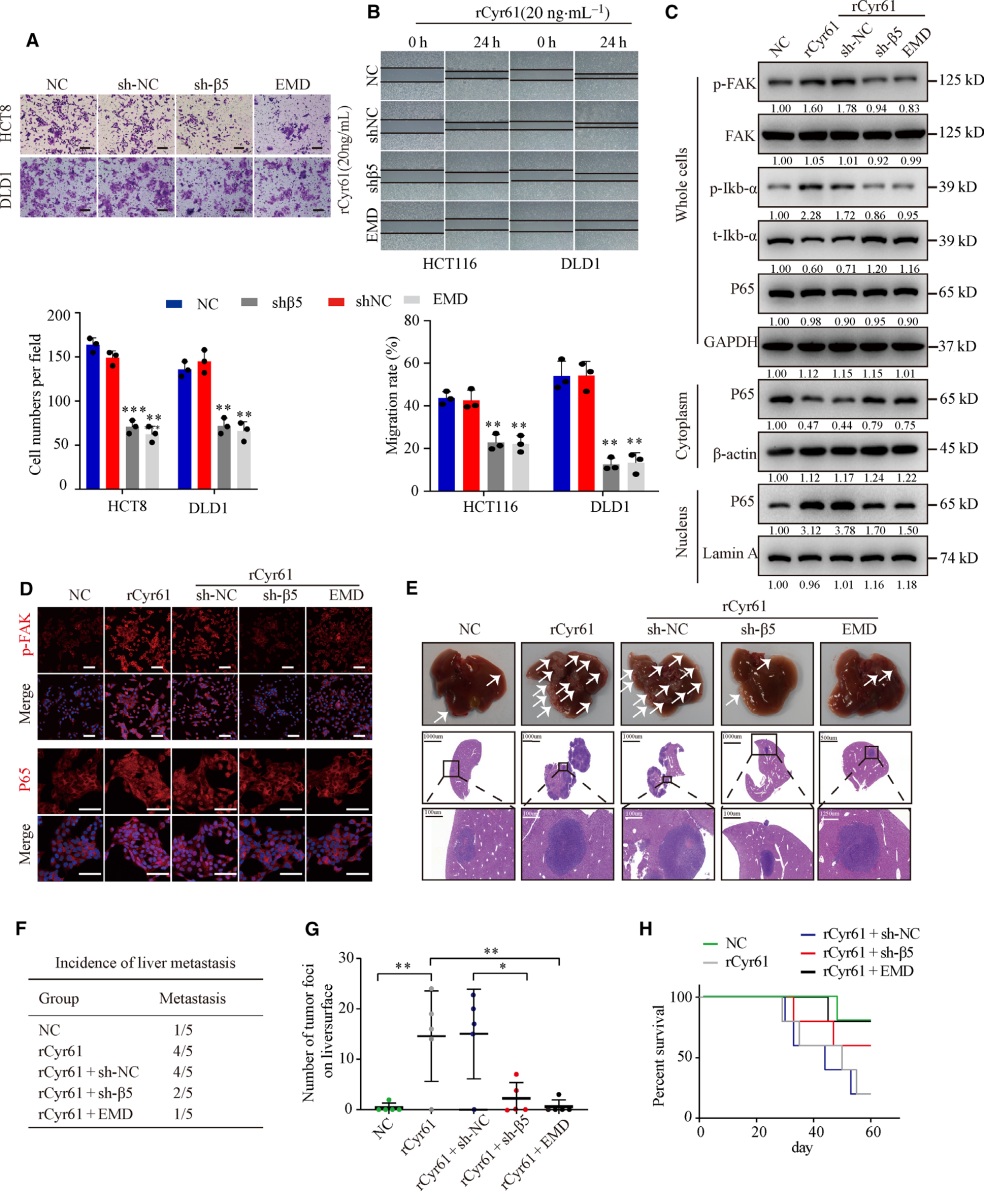
Cyr61 promotes CRC cell migration and invasion *via* α_V_β_5_/FAK/NF‐κB signaling pathway. (A) Representative images of transwell migration assays for HCT8 and DLD1 cells with integrin β_5_ knockdown or treated with integrin α_V_β_5_ inhibitor EMD 121974 (EMD) before treated with rCyr61 (20 ng·mL^−1^). Scale bar = 100 μm, *n* = 3. (B) Representative images of wound‐healing assays for HCT116 and DLD1 cells with integrin β_5_ knockdown or treated with integrin α_V_β_5_ inhibitor EMD before treated with rCyr61 (20 ng·mL^−1^). *n* = 3. (C) Western blot analysis the expression of p‐FAK, FAK, p‐Ikb‐α, t‐Ikb‐αP65, and P65 in HCT8 cells with integrin β_5_ knockdown or with integrin α_V_β_5_ inhibitor EMD. GAPDH, β‐actin, and Lamin A were used as the controls. (D) Confocal microscopy assays were performed to detect the expression of p‐FAK and subcellular localization of P65 in HCT8 cells with integrin β_5_ knockdown or treated with integrin α_V_β_5_ inhibitor EMD. Scale bar = 50 μm. (E) Autopsy and H&E staining of the livers in the nude mice by *in vivo* liver metastasis assays (*n* = 5 per group). The arrows indicated the liver metastasis. Black scale bar = 1000 μm. White scale bar = 100 μm. (F) Incidence of liver metastasis in the nude mice *in vivo* liver metastasis assays. (G) Number of tumor foci on liver surface in the nude mice *in vivo* liver metastasis assays. (H) Kaplan–Meier survival analysis of the nude mice. NC, negative control. Values are represented as mean ± SD. *P < 0.05, **P < 0.01, ***P < 0.001, by two‐tailed Student's *t*‐test and one‐way ANOVA (A, B, G).

To confirm the ability of Cyr61 to promote CRC migration and invasion *via* integrin α_V_β_5_, we employed the integrin α_V_β_5_ inhibitor EMD 121974 (EMD) and integrin β_5_‐specific shRNA (shβ_5_). Compared with the control group, the number of invasive cancer cells was reduced and CRC cells migrated slowly to close the scratched wounds in the presence of EMD or shRNA (Fig. 4A,B). Aberrant nuclear factor‐κB (NF‐κB) activation promotes cancer invasion and metastasis in many cancers, including CRC [37, 38]. As FAK activates a number of downstream molecules, including NF‐κB [39], we hypothesized that Cyr61 binds to integrin α_V_β_5_ to activate the FAK‐NF‐κB signaling pathway to promote CRC cell migration and invasion. As expected, western blot analysis showed that the protein expression of p‐FAK and p‐P65 increased following incubation of HCT8 and DLD1 cells with rCyr61 as determined (Fig. 4C and Fig. S4A). Treatment with shβ_5_ or EMD abrogated the Cyr61‐induced α_V_β_5_/FAK/NF‐κB signaling (Fig. 4C and Fig. S4A). Further analysis of the protein levels of p‐FAK and subcellular localization of P65 by IF revealed upregulated p‐FAK expression and intense nuclear staining of P65 following Cyr61 stimulation. Thus, our results indicated that α_V_β_5_/FAK/NF‐κB signaling pathway is inhibited by shβ5 or EMD (Fig. 4D and Fig. S4B).

Next, we performed *in vivo* metastasis assays by intrasplenic injection of nude mice with HCT8 cells pretreated with rCyr61, shβ_5_, or EMD (*n* = 5 per group). Incubation of HCT8 with rCyr6 increased the number of metastatic nodules in the liver and decreased the survival time compared to the control group. However, shRNA‐mediated knockdown of integrin β_5_‐ or EMD‐induced inhibition of the receptor decreased the number of metastatic nodules and increased the survival time compared to the Cyr61 group (Fig. 4E–H). These findings suggested that Cyr61 promotes CRC cell migration and invasion *via* the integrin α_V_β_5_/FAK/NF‐κB signaling pathway *in vitro* and *in vivo*.

### 3.5 Cyr61 promotes VM formation to promote CRC growth and metastasis

**Fig. 5:**
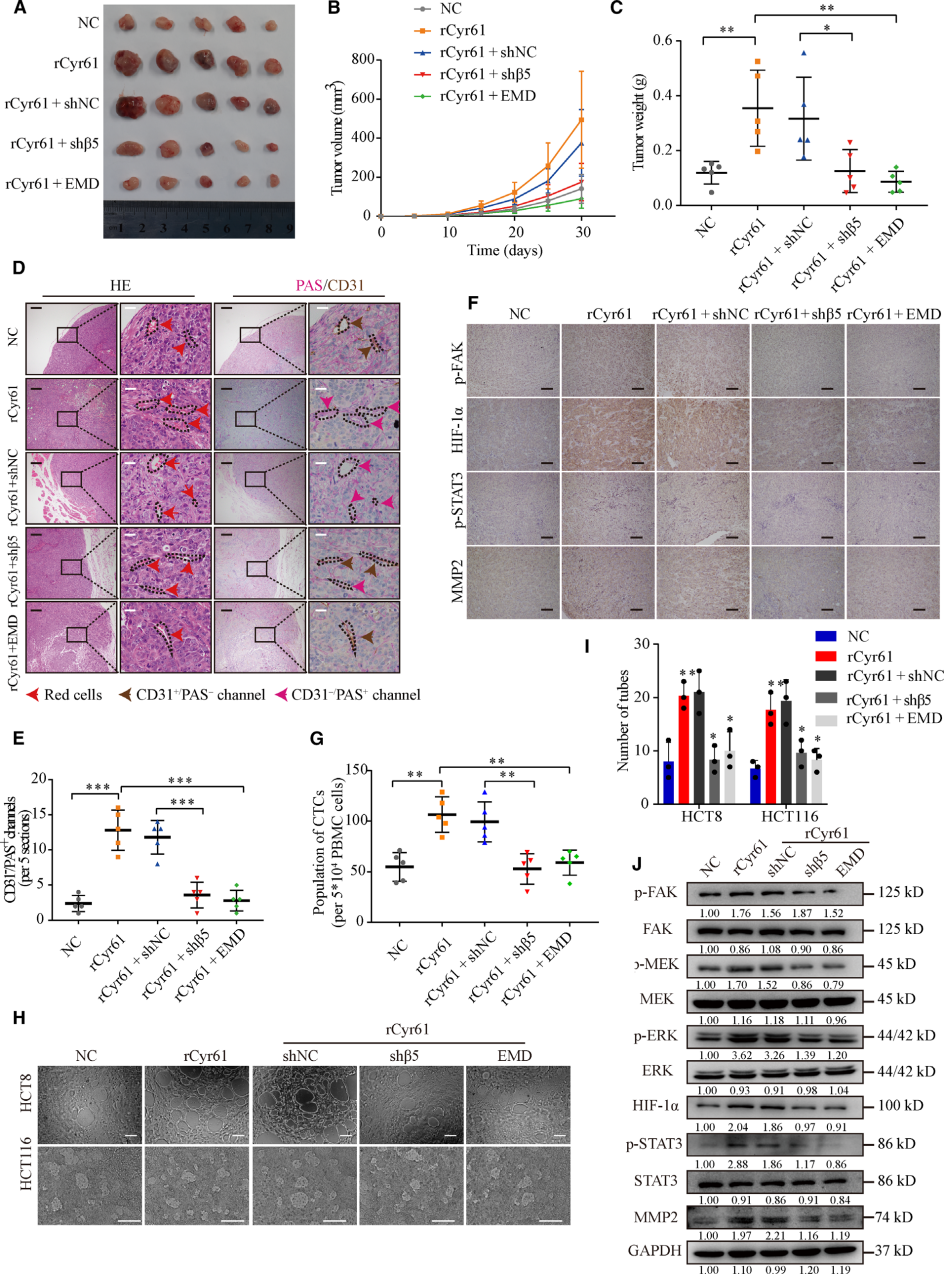
Cyr61 promotes VM formation to promote CRC progression *via* integrin α_V_β_5_. (A) Tumors in nude mice subcutaneously injected with HCT8 cells pretreated with rCyr61, EMD, or knockdown integrin β_5_ expression (*n* = 5 per group). (B) Growth curves of the xenograft tumors. (C) Tumor weight of the HCT8 xenograft tumors. (D) H&E, PAS, and CD31 staining in xenograft tumors. Red arrows indicate the presence of red blood cells; brown arrows indicate typical blood vessels with CD31^+^ staining; pink arrows indicate PAS^+^/CD31^−^ VM channels. Black scale bars = 200 μm, white scale bars = 20 μm. (E) The numbers of PAS^+^/CD31^−^ VM channels in xenograft tumors. (F) IHC analysis of p‐FAK, HIF‐1α, p‐STAT3, and MMP2 expression in xenograft tumors. Scale bars = 100 μm. (G) Statistics of the flow cytometric analysis of the GFP labeled CTCs from whole blood in the nude mice. (H, I) Representative images and statistics of tube formation in HCT8 and HCT116 cells pretreated with rCyr61, EMD, or knockdown integrinβ_5_ expression. Scale bars = 100 μm, *n* = 3. (J) Western blot analysis of α_V_β_5_/FAK/HIF‐1α/STAT3/MMP2 signaling cascade in HCT8 cells pretreated with rCyr61, EMD, or knockdown integrin β5 expression. NC, negative control. Values are represented as mean ± SD. *P < 0.05, **P < 0.01, ***P < 0.001, by 2‐tailed Student’s *t*‐test and one‐way ANOVA (C, E, G, I).

Our previous study indicated that Cyr61 did not influence CRC cell proliferation *in vitro* (Fig. S2E,F). We performed subcutaneous xenograft assays to further confirm this effect *in vivo* (*n* = 5 per group). Intriguingly, the results suggested that Cyr61 increased the volume and weight of tumors. Treatment with shβ_5_ or EMD abrogated the effect of Cyr61 on tumor growth (Fig. 5A–C). A previous study indicated that Epstein–Barr virus‐infected cancer cells promoted VM formation by upregulating the expression of some genes, including Cyr61 [40]. Therefore, we hypothesized that Cyr61 promotes CRC growth by promoting VM formation. The criteria of VM formation were positive for periodic acid‐Schiff (PAS) but negative for CD31 (PAS^+^/CD31^−^) and the existence of erythrocytes in the vascular‐like channels [41]. The results showed that tumors derived from cells treated with rCyr61 exhibited more VM structures. As expected, treatment with shβ_5_ or EMD decreased the number of VM structures (Fig. 5D,E).

The molecular mechanism of VM formation was complicated, in which HIF‐1α and MMP2 play critical roles [40, 42]. Furthermore, p‐STAT3 upregulated MMP2 expression, which promoted the formation of VM [42]. In previous experiments, we identified that Cyr61 activates the FAK signaling pathway (Fig. 4C,D). Since FAK activation can stimulate the FAK/MEK/ERK signaling pathway to activate transcription factors [43], we hypothesized that Cyr61 promotes VM formation *via* the α_V_β_5_/FAK signaling cascade to activate HIF‐1α and MMP2. We then verified this hypothesis in subcutaneous tumors, and the results suggested that these two transcription factors were active after rCyr61 treatment. Consistently, shβ5 or EMD treatment suppressed this signaling cascade (Fig. 5F and Fig. S5A). Emerging evidence indicated that VM was related to clinical stage [44] and tumor metastasis [45], with a relationship between VM and the existence of CTCs [46]. Flow cytometric analysis of CTCs in whole‐blood samples from the mice showed that treatment with rCyr61 increased the number of CTCs compared to the control group. Conversely, treatment with shβ5 or EMD decreased the number of CTCs (Fig. 5G and Fig. S5B).

Next, we applied a 3D culture system to identify VM formation *in vitro*. HCT8 and HCT116 cells treated with rCyr61 showed vessel‐like structures, while CRC cells treated with shβ5 or EMD spread evenly on the matrix surface in a pattern that was similar to that observed in the control group (Fig. 5H,I). Moreover, western blot analysis was performed to confirm the ability of Cyr61 to activate HIF‐1α and MMP2 *in vitro*. As expected, the protein level of HIF‐1α and MMP2 were increased *via* the FAK/MEK/ERK signaling activation pathway (Fig. 5J and Fig. S5C). We further analyzed the protein levels of integrin α_V_β_5_/FAK/HIF‐1α/STAT3/MMP2 signaling cascade *via* IF assays. Compared with the controls, IF assays revealed upregulated expression of p‐FAK, p‐MEK, and MMP2 and intense nuclear staining of ERK, HIF‐1α, and STAT3 in CRC cells treated with rCyr61 (Fig. S5D). Taken together, these results suggested that Cyr61 enhances VM formation to promote CRC growth and metastasis *in vivo* and *in vitro* *via* the integrin α_V_β_5_/FAK/HIF‐1α/STAT3/MMP2 signaling cascade.

### 3.6 Cyr61 is related to VM formation in CRC tissues

**Fig. 6:**
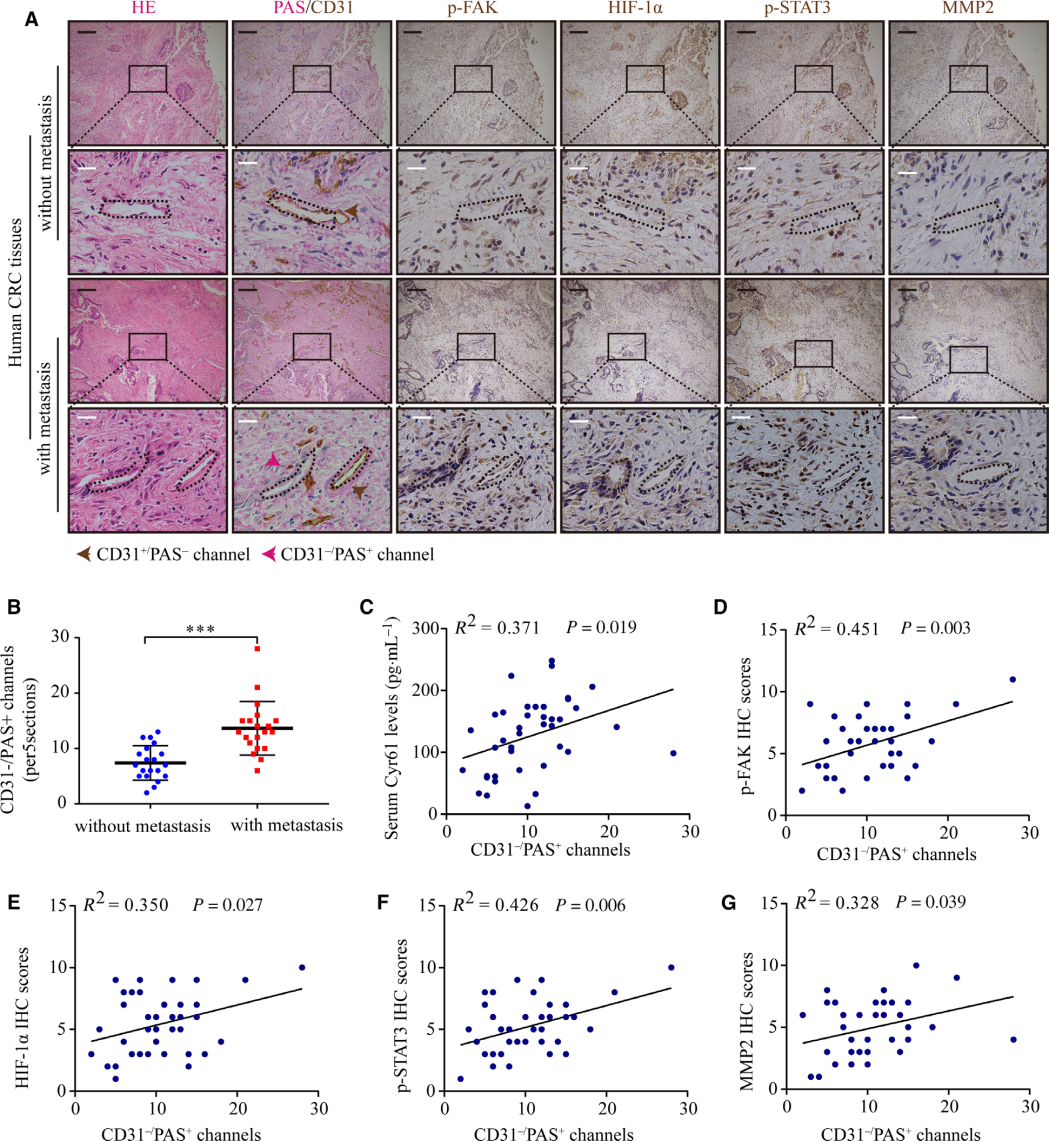
The relationship between serum Cyr61 levels and VM in CRC tissues. (A) H&E staining, VM formation, p‐FAK, HIF‐1α, p‐STAT3, and MMP2 expression in CRC patients with or without metastasis. *n* = 20 per group. Black scale bars = 200 μm; white scale bars = 20 μm. (B) Statistics of PAS^+^/CD31^−^ VM channels in CRC patients with or without metastasis. (C–G) Pearson correlation of PAS^+^/CD31^−^ VM channels with serum Cyr61 levels, p‐FAK, HIF‐1α, p‐STAT3, and MMP2 IHC scores in CRC tissues. Values are represented as mean ± SD. ***P < 0.001, by two‐tailed Student's *t‐*test (B).

We further investigated VM formation and the expression levels of proteins related to the FAK/HIF‐1α/STAT3/MMP2 signaling cascade in CRC tissues with or without metastasis. Forty cases of CRC tissues, half of which were with metastasis, were selected from 364 CRC patients whose serum Cyr61 protein levels had been analyzed before. Compared with the cases without metastasis, we found that the number of PAS^+^/CD31^−^ vascular‐like channels increased in CRC tissues with metastasis (Fig. 6A,B). IHC analysis showed that the protein levels of p‐FAK, HIF‐1α, p‐STAT3, and MMP2 were also upregulated in CRC tissues with metastasis (Fig. 6A). Moreover, the numbers of PAS^+^/CD31^−^ vascular‐like channels correlated positively with serum Cyr61 protein levels and the levels of p‐FAK, HIF‐1α, p‐STAT3, and MMP2 protein in tissues (Fig. 6C–G). Thus, these data indicated the existence of VM in CRC tissues and further confirmed the association between VM formation, metastasis, and activation of the FAK/HIF‐1α/STAT3/MMP2 signaling cascade.

### 3.7 Synergistic effect of anti‐VM by integrin αVβ5 inhibitor EMD and anti‐VEGF by bevacizumab therapy in CRC PDX models

**Fig. 7:**
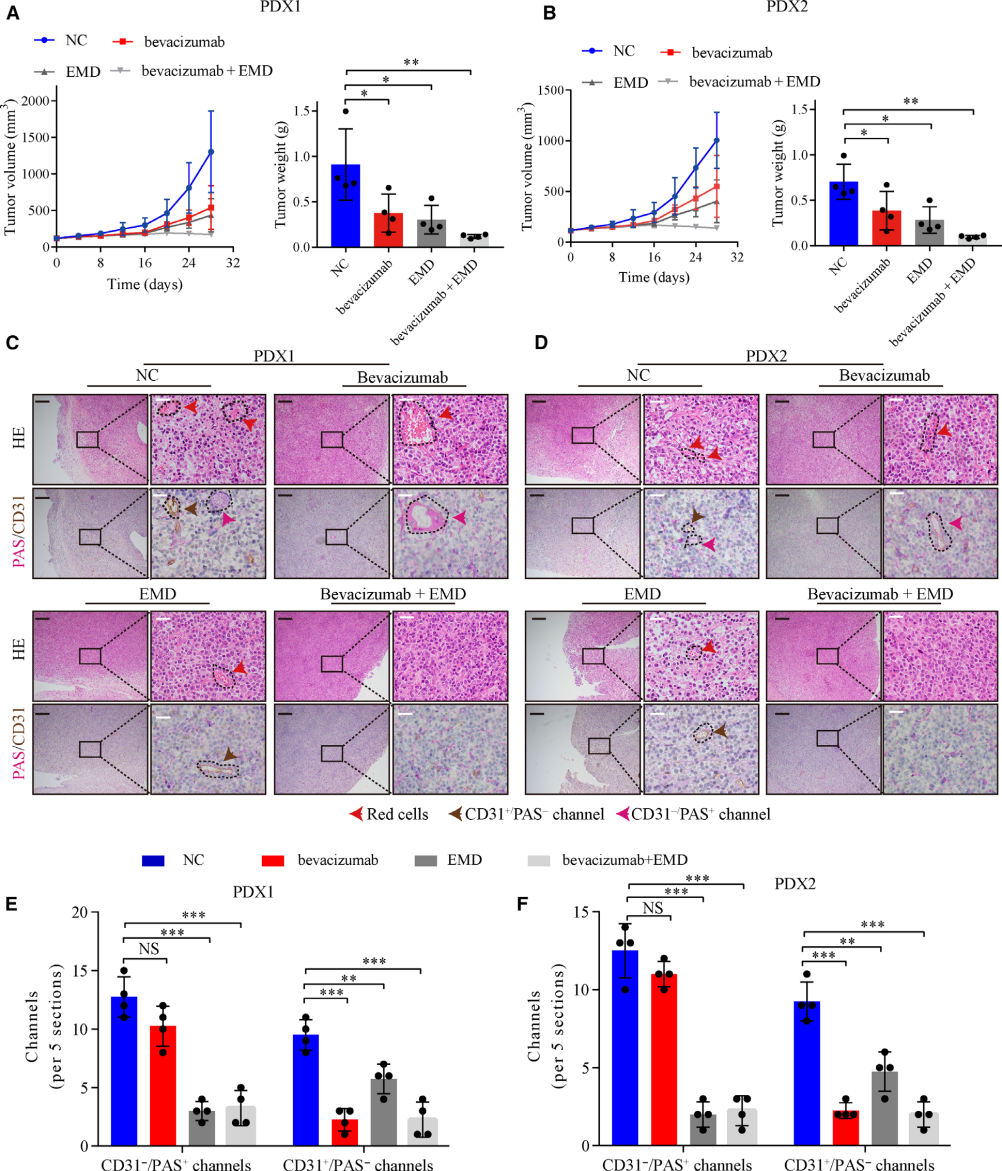
Synergistic effect of EMD and bevacizumab in CRC PDX models. (A, B) Growth curves and tumor weight of PDX model tumors after treatment with vehicle, EMD, or/and bevacizumab. (*n* = 4) (C, D) H&E, PAS, and CD31 staining in PDX model tumors after treatment with vehicle, EMD, or/and bevacizumab. Black scale bars = 200 μm; white scale bars = 20 μm, *n* = 4. (E, F) Statistics of PAS^+^/CD31^−^ VM channels and PAS^−^/CD31^+^ channels in PDX model tumors after treatment with vehicle, EMD or/and bevacizumab. Values are represented as mean ± SD. NC, negative control. NS, no significant, *P < 0.05, **P < 0.01, ***P < 0.001, by one‐way ANOVA (A, B, E, F).

Since angiogenesis plays a vital role in CRC growth and metastasis, anti‐VEGF therapy is a useful treatment strategy [47]. PDX models retain the characteristics of the original cancer and are used for curative effect analysis and preclinical drug evaluation [48]. Therefore, we analyzed the synergistic effect of integrin α_V_β_5_ inhibitor EMD and bevacizumab, a wildly used anti‐VEGF monoclonal antibody, in two PDX models. Treatment with EMD or bevacizumab alone inhibited tumor growth moderately compared to the control group. Notably, the combination of EMD and bevacizumab significantly inhibited tumor growth (Fig. 7A,B). Next, we analyzed the number of VM channels and microvessels in PDX tumors by double staining of PAS and CD31. Treatment with bevacizumab decreased the number of CD31^+^ microvessels. Furthermore, EMD treatment significantly decreased the number of PAS^+^/CD31^−^ channels and moderately decreased the number of CD31^+^ microvessels. Combination treatment with EMD and bevacizumab markedly decreased both channel formation (Fig. 7C–F). Taken together, these data suggested that combination of anti‐VM by EMD and anti‐VEGF by bevacizumab was a novel therapeutic strategy for CRC.

### 3.8 CRC cell‐derived exosomal STAT3 promotes Cyr61 transcription

**Fig. 8:**
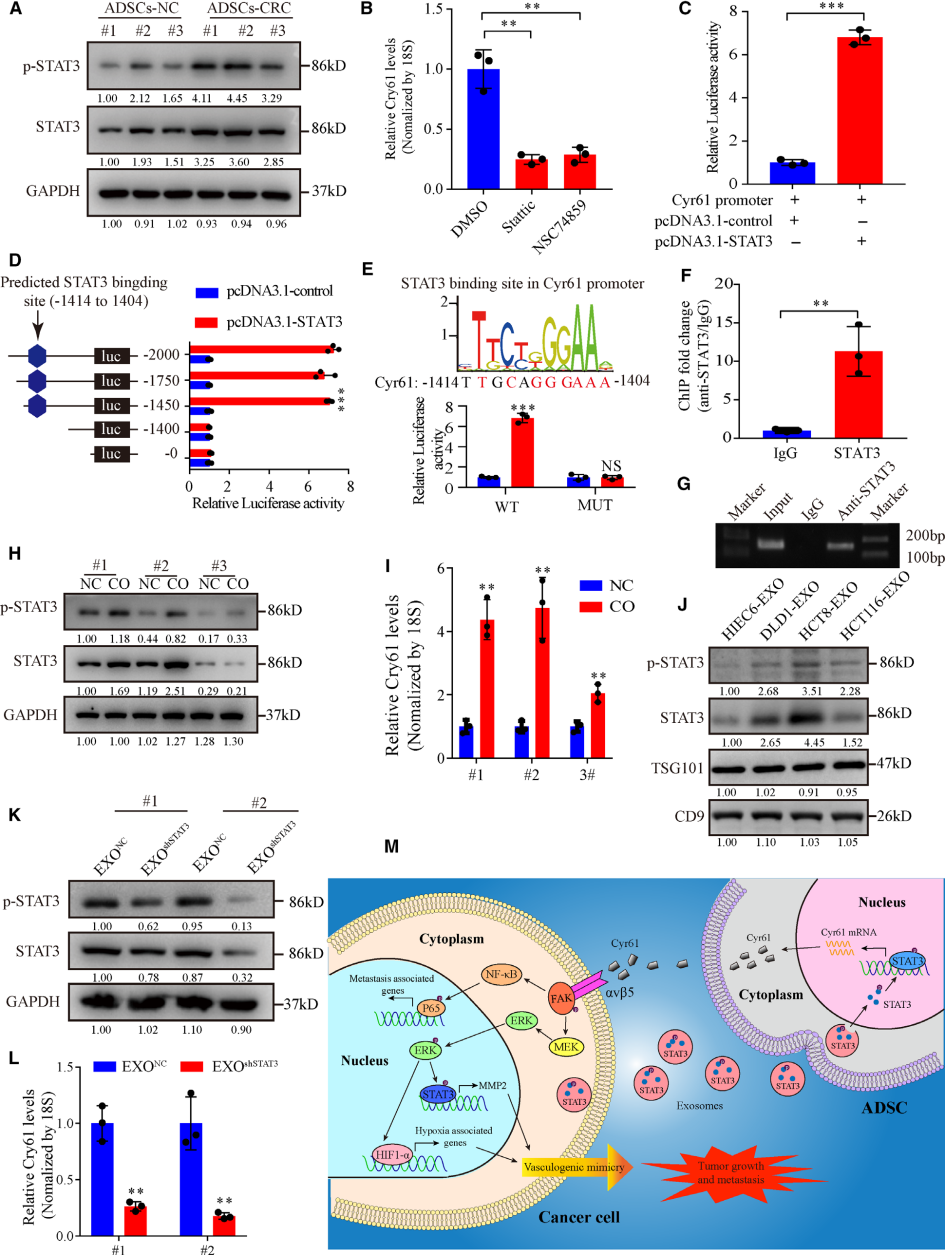
CRC cell‐derived exosomal STAT3 promotes Cyr61 transcription. (A) Western blot analysis of p‐STAT3 and STAT3 expression in ADSCs‐NC and ADSCs‐CRC. (B) qRT‐PCR analysis of Cyr61 mRNA levels in ADSCs treated with STAT3 inhibitors. *n* = 3. (C) Luciferase activity of the Cyr61 promoter in ADSCs transfected with STAT3. *n* = 3. (D) Luciferase (luc) reporter assays for ADSCs transfected with a series of reporter vectors containing sequential deletion of the Cyr61 promotor. *n* = 3. (E) Potential STAT3‐binding site in the Cyr61 promoter predicted in the JASPAR database. Luciferase reporter assays for ADSCs transfected with reporter plasmids containing point mutation at the STAT3‐binding site. *n* = 3. (F, G) qRT‐PCR analysis of the product of chromatin immunoprecipitation assays for the binding of STAT3 to the crucial STAT3‐binding site in the Cyr61 promoter. (H) Western blot analysis of p‐STAT3 and STAT3 expression changes in ADSCs cocultured with HC8 cells. (I) qRT‐PCR analysis of Cyr61 mRNA levels in ADSCs cocultured with HC8 cells. *n* = 3. (J) Western blot analysis of p‐STAT3 and STAT3 in normal colonic cell and CRC cell‐derived exosomes. (K) Western blot analysis of p‐STAT3 and STAT3 expression in ADSCs cocultured with CRC cell‐derived exosomes with or without p‐STAT3 and STAT3 knockdown. (L) qRT‐PCR analysis of Cyr61 mRNA levels in ADSCs cocultured with CRC cell‐derived exosomes with or without p‐STAT3 and STAT3 knockdown. *n* = 3. (M) Schematic model of the role of Cyr61 in CRC tumor progression. Values are represented as mean ± SD. CO, cocultured; EXO, exosomes. NS, no significant, **P < 0.01, ***P < 0.001, by two‐tailed Student's *t*‐test (C, E, F, I, L) and one‐way ANOVA (B, D).

We finally investigated the potential mechanisms underlying the increased secretion of Cyr61 in ADSCs isolated from CRC patients. It had been reported that Cyr61 is a target gene of STAT3 in embryonic stem cells [49]. Therefore, we speculated that STAT3 plays a vital role in Cyr61 transcription in ADSCs. Western blot analysis showed that the protein levels of STAT3 and p‐STAT3 were increased in ADSCs‐CRC compared to ADSCs‐NC (Fig. 8A). More importantly, STAT3 inhibitors suppressed Cyr61 RNA levels significantly (Fig. 8B). Next, we cloned a fragment containing the Cyr61 promotor into a luciferase reporter vector and the results indicated that STAT3 markedly increased the luciferase activity (Fig. 8C). The potential STAT3‐binding site (−1414 to −1404 bp) was predicted by the JASPAR database. To identify the binding site, a series of reporter vectors containing sequential deletions of the Cyr61 promotor were transfected into ADSCs. As shown in Fig. 8D, the −1414 to −1404 bp region was identified as the potential STAT3‐binding site. Mutation of four nucleotides at the STAT3‐binding site decreased the luciferase activity (Fig. 8E). ChIP assays were performed to further verify the direct interaction between STAT3 and the −1414 to −1404 bp region (Fig. 8F,G). Altogether, these data suggested that STAT3 is involved in Cyr61 transcription.

Next, we investigated the mechanism by which STAT3 and p‐STAT3 are upregulated in ADSCs‐CRC and the potential influence of tumor cells. Cocultured of ADSCs with CRC cells resulted in increased protein levels of STAT3 and p‐STAT3 (Fig. 8H) and increased Cyr61 RNA levels (Fig. 8I). Accumulating evidence suggests that exosomes play a vital role in cell‐to‐cell communication through transfer of exosomal contents [50]. We identified the existence of STAT3 and p‐STAT3 proteins in exosomes extracted from the culture medium of normal colonic cell and three CRC cell lines (Fig. 8J). Furthermore, exosomal STAT3 and p‐STAT3 levels in STAT3 knockdown HCT8 cells were consistent with those changes in the parental cells (Fig. S6A,B). To further identify the function of tumor‐derived exosomal STAT3 and p‐STAT3 in ADSCs, we cocultured ADSCs with these exosomes. We found the protein levels of STAT3 and p‐STAT3 and the RNA levels of Cyr61 were decreased when ADSCs were cocultured with EXO^shSTAT3^ (Fig. 8K,L). These findings suggested that CRC cell‐derived exosomal STAT3 and p‐STAT3 play a vital role in Cyr61 transcription.

## 4 Discussion

Cyr61, also known as CCN1, has diverse biological functions, such as promoting cell migration and proliferation by binding to cell‐specific integrin receptors [22]. A previous study also showed that Cyr61 was upregulated in the serum of CRC patients [21]. However, the source of serum Cyr61 and the mechanisms by which Cyr61 promotes CRC progression still remain unknown. In this study, we found that CRC‐associated ADSCs secreted more Cyr61 than ADSCs‐NC and the serum Cyr61 levels were associated with advanced TNM stages. Mechanistically, Cyr61 promoted CRC cell metastasis *in vitro* and *in vivo* by activating integrin α_V_β_5_. In addition, Cyr61 could promote VM formation to promote CRC progression *via* integrin α_V_β_5_. Moreover, a synergistic effect of anti‐VM by integrin α_V_β_5_ inhibitor and anti‐VEGF by bevacizumab therapy was identified in PDX models. These findings indicated that Cyr61 derived from ADSCs plays a critical role in promoting CRC progression *via* integrin α_V_β_5_ and provided a novel antitumor strategy.

Instead of canonical RGD sequence, Cyr61 contained the noncanonical RGD sequence that mediates binding to various integrins [51]. The interaction of Cyr61 with different integrin receptors contributes to their specific functions. Cyr61 interacts with integrin α_v_β_3_, α_v_β_5_, and α_6_β_1_ to stimulate fibroblasts cell DNA synthesis, adhesion, and migration, respectively [52]. Cyr61 stimulates endothelial cell and vascular smooth muscle cell migration by binding to α_v_β_3_ and α_6_β_1_ [23]. Mutational analysis indicated that Cyr61 function by binding to specific integrin receptors independently of one another [53]. In this study, we demonstrated that serum Cyr61 was derived mainly from ADSCs rather than the tumor itself. Serum Cyr61 exhibited a better diagnostic value (AUC = 0.933) for CRC compared to CEA and CA199, suggesting that serum Cyr61 exhibits higher sensitivity and specificity as a diagnostic marker. Thus, we propose that plasma Cyr61 is a new promising new biomarker for the diagnosis and prognosis of CRC. Moreover, we identified integrin α_V_β_5_ as the functional receptor of Cyr61 on CRC cells and showed that Cyr61 interacted directly with integrin α_V_β_5_ to activate the downstream FAK signaling pathway to promote CRC progression.

Vasculogenic mimicry, a vascular channel‐like structure that consists of tumor cells but not ECs, was first reported in in uveal melanoma in 1999 [54]. VM formation is identified by positive PAS staining in the absence of CD31 expression and red blood cells in the vascular‐like channels [41]. VM has been identified in various malignant tumors, including CRC [55]. Hypoxic tumor microenvironment was a common phenomenon because of vast oxygen consuming [56]. Tumor microenvironment becomes hypoxic increasingly with tumor growth. Under hypoxic condition, cancer cells undergo adaptive changes, such as HIF‐1α activation. HIF‐1α and MMP2 play critical roles in VM formation [40, 42]. Previous studies demonstrated that STAT3 blocks HIF‐1α degradation and accelerates its de novo synthesis to enhance its stability and activity [42]. In addition, p‐STAT3 upregulates MMP2 expression to promote the formation of VM [42]. In this study, the *in vitro* experiments indicated that CRC cell proliferation was not influenced by Cyr61, which seemed to be inconsistent with the subcutaneous xenografts assays which showed that Cyr61 increased the volume and weight of tumors (Fig. 5A–C). This can be explained by the findings that Cyr61 binds to its functional receptor integrin α_v_β_5_ to activate FAK signaling pathway, thereby upregulating the expression of HIF‐1α and MMP2, and ultimately stimulating VM formation to promote CRC cell proliferation *in vivo*.

Anti‐ angiogenesis is a promising treatment in CRC. Bevacizumab, a widely used anti‐VEGF monoclonal antibody, has been evaluated in a multicenter phase II clinical trial in CRC patients with metastasis [57]. However, anti‐angiogenesis with VEGF inhibitors causes a hypoxic tumor microenvironment that may promote tumor progression through VM formation. Therefore, anti‐VM therapy combined with anti‐VEGF to block the supply of oxygen and nutrition to cancer cells is a strategy to overcome the side effect of anti‐VEGF therapy. In this study, we found that Cyr61 promoted VM formation by binding to integrin α_V_β_5_. The integrin α_V_β_5_ inhibitor cilengitide (EMD) has been evaluated in clinical trials in non‐small‐cell lung cancer and glioblastoma [58, 59]. Combination treatment with EMD and bevacizumab decreased the number of VM channels and microvessels in CRC PDX models. This indicated the potential of cilengitide for clinical application in CRC treatment. In addition to integrin α_V_β_5_, targeting serum Cyr61 or the integrin α_V_β_5_/FAK signaling pathway may also decrease tumor progression.

## 5 Conclusions

Our findings revealed that ADSC‐derived Cyr61 promotes CRC metastasis and VM formation *via* its functional receptor integrin α_V_β_5_ to activate the FAK signaling pathway (Fig. 8M). Moreover, combination therapy with cilengitide and bevacizumab is implicated as a potential novel antitumor strategy for CRC.

## Conflict of interest

All authors declare no conflict of interests.

## Author contributions

ZL performed *in vitro* experiments; ZL and HL performed animal study together; YZ and XW performed the ISH scoring analyses; YZ, ZZ, FW, and XH helped to organize and analyze the data; ZL drafted this manuscript and PL and XW corrected it; PL and XW supervised this study and provided funding.

### Peer Review

The peer review history for this article is available at https://publons.com/publon/10.1002/1878‐0261.12998.

## Supporting information


**Fig. S1**. Characterization of ADSCs.
**Fig. S2**. ADSCs derived Cyr61 have no effect on CRC cell proliferation *in vitro*.
**Fig. S3**. Cyr61 receptor identification on CRC cells.
**Fig. S4**. The α_V_β_5_/FAK/NF‐κB signaling pathway changes in DLD1 cells.
**Fig. S5**. The α_V_β_5_/FAK/HIF‐1α/STAT3/MMP2 signaling cascade changes after Cyr61 treatment.
**Fig. S6**. Western blot analysis of the efficiency of shRNA for STAT3.
**Table S1**. Correlation between Cyr61 levels and clinicopathologic characteristics of CRC patients.
**Table S2**. The ROC curve assay of CEA, CA199, CA125 and Cyr61.
**Table S3**. List of surface membrane proteins of mass spectrometry results (score>35).
**Table S4**. Oligonucleotide sequences.

## Data Availability

The mass spectrometry data have been deposited to the ProteomeXchange Consortium *via* the PRIDE [60] partner repository with the dataset identifier PXD019954.
