# Supplementary material for: Cyr61 from adipose‐derived stem cells promotes colorectal cancer metastasis and vasculogenic mimicry formation via integrin αVβ5
Source: Mol Oncol. 2021 Jun 1;15(12):3447–67. doi: 10.1002/1878-0261.12998 (PMC8637569; doi:10.1002/1878-0261.12998)
Supplement: Supplementary file 1 — Fig. S1. Characterization of ADSCs. Fig. S2. ADSCs derived Cyr61 have no effect on CRC cell proliferation in vitro. Fig. S3. Cyr61 receptor identification on CRC cells. Fig. S4. The αVβ5/FAK/NF‐κB signaling pathway changes in DLD1 cells. Fig. S5. The αVβ5/FAK/HIF‐1α/STAT3/MMP2 signaling cascade changes after Cyr61 treatment. Fig. S6. Western blot analysis of the efficiency of shRNA for STAT3. Table S1. Correlation between Cyr61 levels and clinicopathologic characteristics of CRC patients. Table S2. The ROC curve assay of CEA, CA199, CA125 and Cyr61. Table S3. List of surface membrane proteins of mass spectrometry results (score>35). Table S4. Oligonucleotide sequences. [file MOL2-15-3447-s001.pdf]

Title: Cyr61 from adipose-derived stem cells promotes colorectal cancer metastasis and vasculogenic mimicry formation via integrin $\alpha_V\beta_5$

Authors: Zhenxing Liang[1, 2, 3], Huashan Liu[1, 2, 3], Yunfeng Zhang[1], Li Xiong[4], Ziwei Zeng[1], Xiaowen He[1], Fengwei Wang[5], Xianrui Wu[1, 2, 3] and Ping Lan[1, 2, 3]

From: [1]Department of Colorectal Surgery, The Sixth Affiliated Hospital, Sun Yat-sen University, Guangzhou, Guangdong, China.

[2]Guangdong Provincial Key Laboratory of Colorectal and Pelvic Floor Diseases, The Sixth Affiliated Hospital, Sun Yat-sen University, Guangzhou, Guangdong, China.

[3]Guangzhou Regenerative Medicine and Health Guangdong Laboratory, Guangzhou, China.

[4]Department of Endocrinology, The First Affiliated Hospital of Sun Yat-sen University, Guangzhou, China.

[5]State Key Laboratory of Oncology in South China, Sun Yat-sen University Cancer Center, Guangzhou, Guangdong, China.

Authorship: Zhenxing Liang and Huashan Liu contributed equally to this study.

Correspondence authors:

Ping Lan and Xianrui Wu

The Sixth Affiliated Hospital, Sun Yat-sen University

26 Yuancun Erheng Rd, Guangzhou, Guangdong, China, 510655

Tel: 086-020-38255801; Fax: 011-86-20-38254166.

Email: lanping@mail.sysu.edu.cn; wuxianr5@mail.sysu.edu.cn

Fengwei Wang

State Key Laboratory of Oncology in South China,

Sun Yat-Sen University Cancer Center,

No. 651, Dongfeng Road East, 510060 Guangzhou, China

Tel: 86-20-87343193; Fax: 86-20-87343170

Email: wangfengw@sysucc.org.cn

Conflict of interest: The authors have declared that no conflict of interest exists.

Supplemental Figures

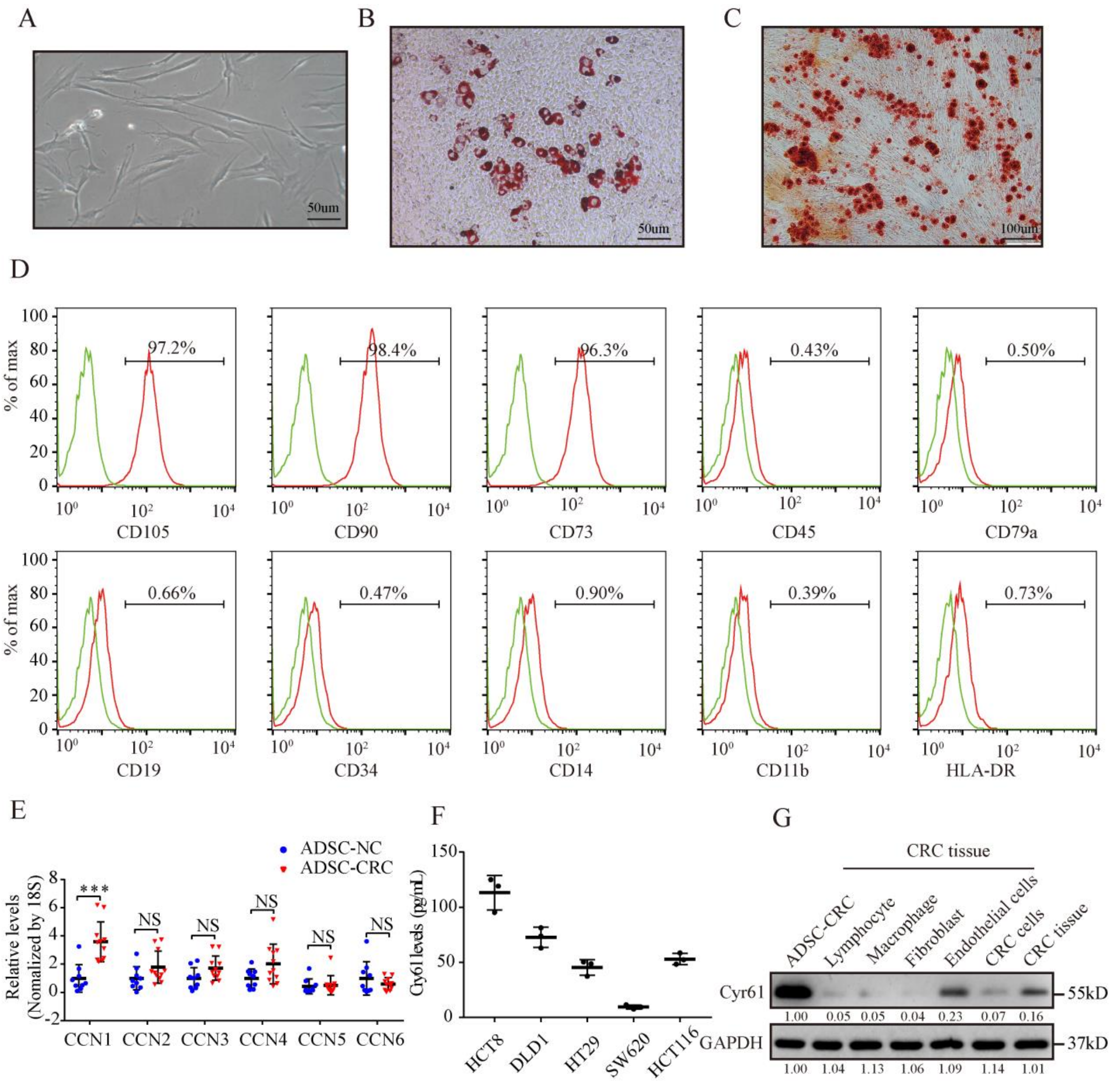

Supplementary Figure 1. Characterization of ADSCs

A Morphology of ADSCs. Scale bar = 50 μm. **B** ADSCs differentiated toward the adipogenic lineages, as evidenced by Oil Red O staining. Scale bar = 50 μm. **C** ADSCs differentiated toward the osteogenic lineages, as evidenced by Alizarin Red S staining. Scale bar = 100 μm. **D** Flow cytometric analysis of the ADSCs surface markers CD105, CD90, CD73, CD45, CD79a, CD19, CD34, CD14, CD11b and HLA-DR. IgG isotypes were used as negative controls. **E** qRT-PCR analysis of CCN protein family mRNA levels in ADSCs-NC (n=9) and ADSCs-CRC (n=11). **F** ELISA analysis of medium Cyr61 levels in CRC cell lines. **G** Western blot analysis of Cyr61 protein levels in ADSC-CRC, CRC tissues, and lymphocyte, macrophage, fibroblast endothelial cells and CRC cells isolated from CRC tissue. Values are represented as

mean ± SD. NS, no significant, ***p < 0.001, by 2-tailed Student's t test (**E**).

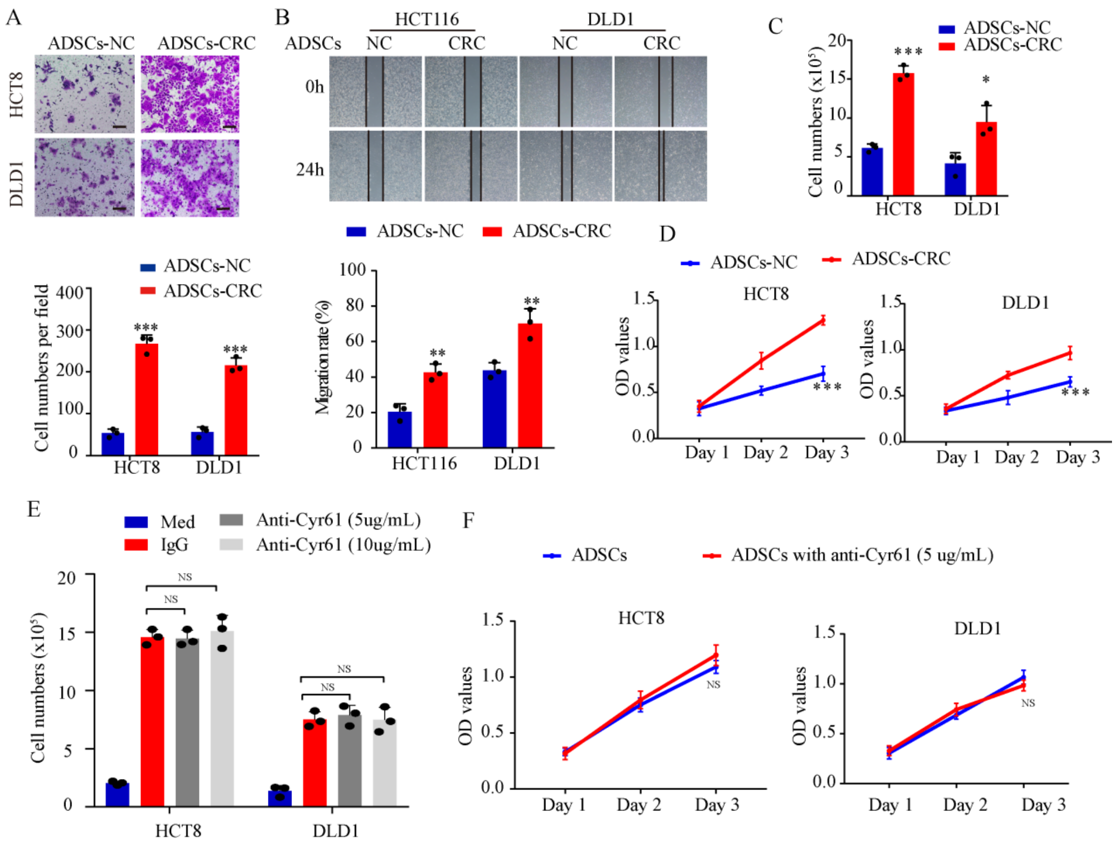

Supplementary Figure 2. ADSCs derived Cyr61 have no effect on CRC cell proliferation *in vitro*

A Representative images of transwell migration assays for HCT8 and DLD1 cells co-cultured with ADSCs-NC or ADSCs-CRC. Scale bar = 100 μm. **B** Representative images of wound-healing assays for HCT116 and DLD1 cells co-cultured with ADSCs-NC or ADSCs-CRC. **C** and **D** Cell counting and MTS assays for HCT8 and DLD1 cells co-cultured with ADSCs-NC or ADSCs-CRC. **E** and **F** Cell counting and MTS assays for HCT8 and DLD1 cells co-cultured with culture medium alone (Med) or ADSCs in the presence or absence of an anti-Cyr61 antibody at 5 or 10μg/mL, or an isotype-matched IgG control (IgG). Values are represented as mean ± SD. NS, no significant, *p < 0.05, **p < 0.01, ***p < 0.001, by 2-tailed Student's t test (**A**, **B**, **C**, **D** and **F**) and one-way ANOVA (**E**).

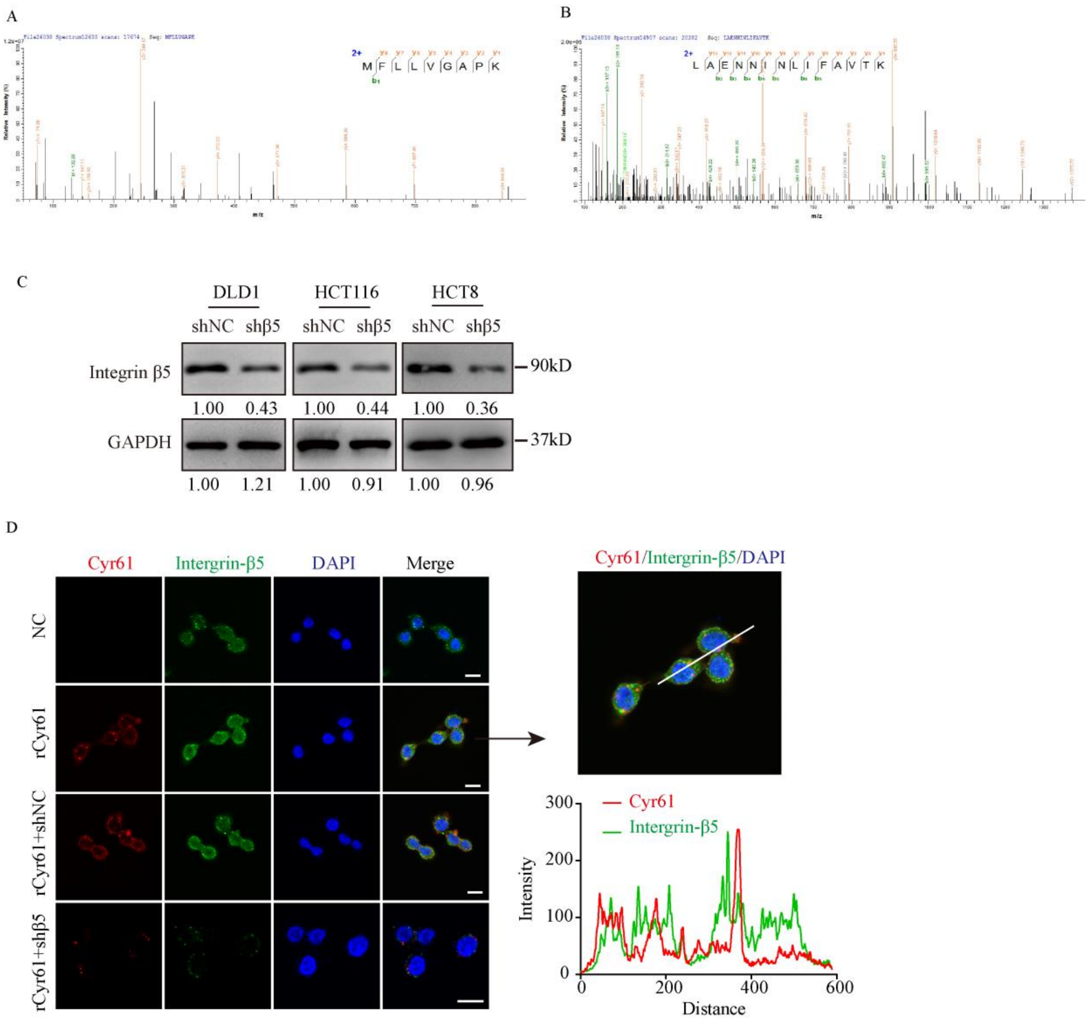

Supplementary Figure 3. Cyr61 receptor identification on CRC cells

A Mass spectra of a representative peptide fragment of integrin α_V from the bands. **B** Mass spectra of a representative peptide fragment of integrin β_5 from the bands. **C** Western blot analysis of the efficiency of shRNA for integrin β_5 in DLD1, HCT116 and HCT8 cells. **D** Confocal microscopy to confirm the colocalization between Cyr61 and integrin $\alpha_V\beta_5$ on HCT8 cells. Scale bar = 10 μm.

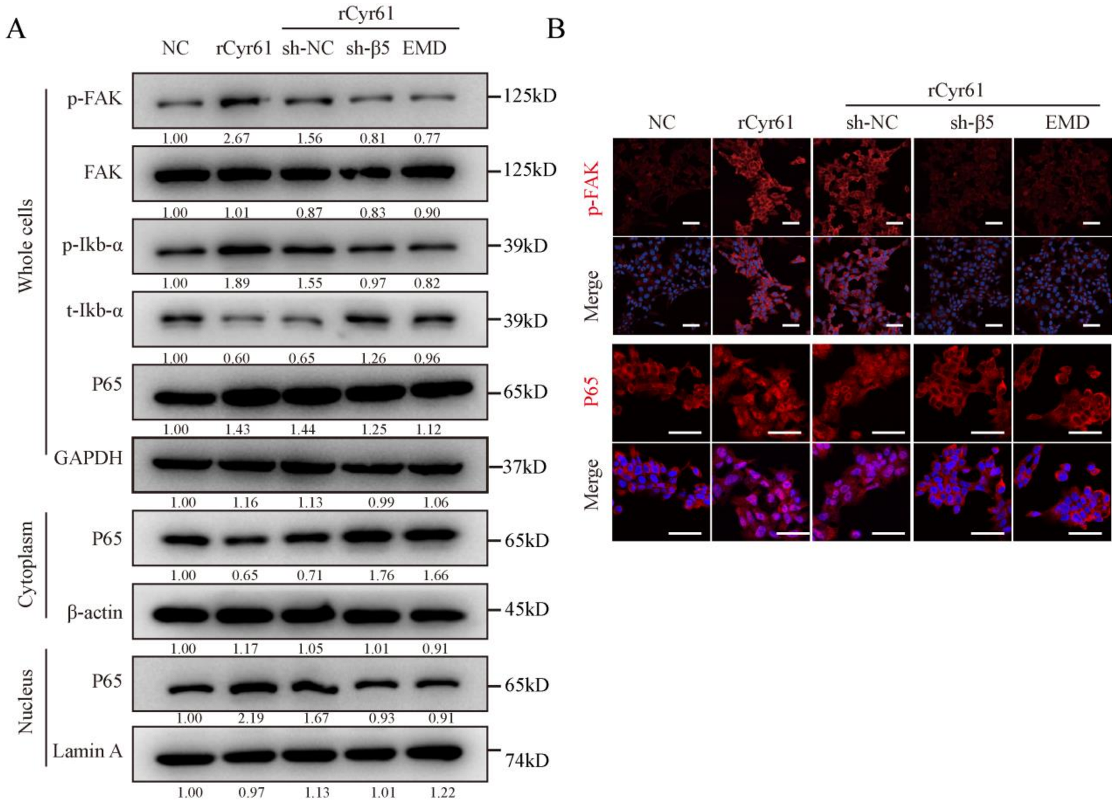

Supplementary Figure 4. The $\alpha_V\beta_5$/FAK/NF-κB signaling pathway changes in DLD1 cells

A Western blot analysis the expression of p-FAK, FAK, p-Ikb-α, t-Ikb-αP65 and P65 in DLD1 cells with integrinβ_5 knockdown or with integrin $\alpha_V\beta_5$ inhibitor EMD. GAPDH, β-actin and Lamin A were used as the controls. **B** Confocal microscopy of DLD1 cells with integrinβ_5 knockdown or treated with integrin $\alpha_V\beta_5$ inhibitor EMD for the expression of p-FAK and subcellular localization of P65. Scale bar = 50 μm.

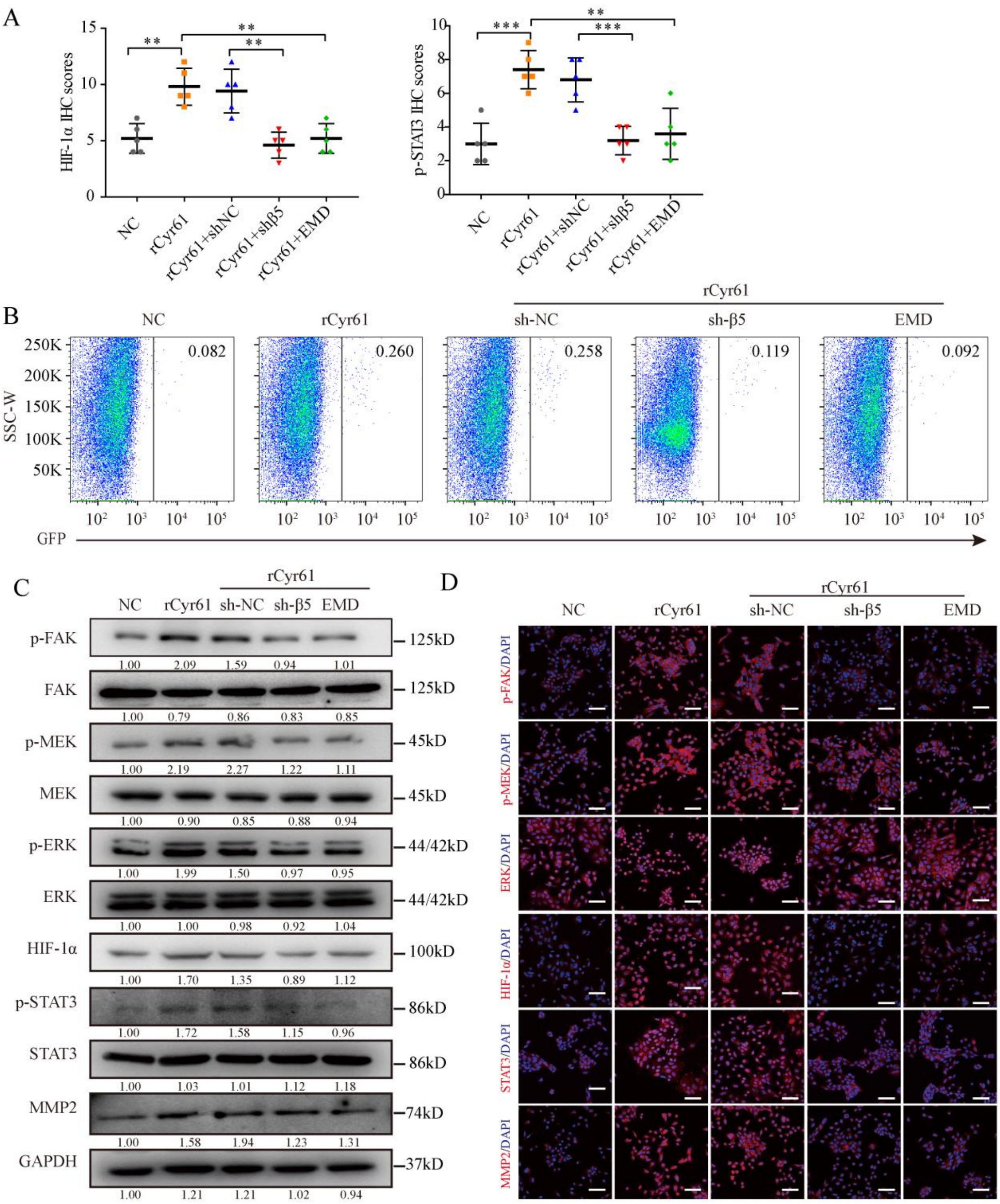

Supplementary Figure 5. The $\alpha_V\beta_5$/FAK/HIF-1α/STAT3/MMP2 signaling cascade changes after Cyr61 treatment

A IHC scoring analyses of p-STAT3 and HIF-1α expression in the subcutaneous tumors. **B** Flow cytometric analysis of the GFP labeled CTCs from the nude mice whole-blood. **C** Western blot analysis of $\alpha_V\beta_5$/FAK/HIF-1α/STAT3/MMP2 signaling cascade in DLD1 cells pretreated with rCyr61, EMD or knockdown integrinβ5 expression. **D** Confocal microscopy analysis of $\alpha_V\beta_5$/FAK/HIF-1α/STAT3/MMP2 signaling cascade in HCT8 cells pretreated with rCyr61, EMD or knockdown

integrinβ_5 expression. Scale bar = 50 μm.

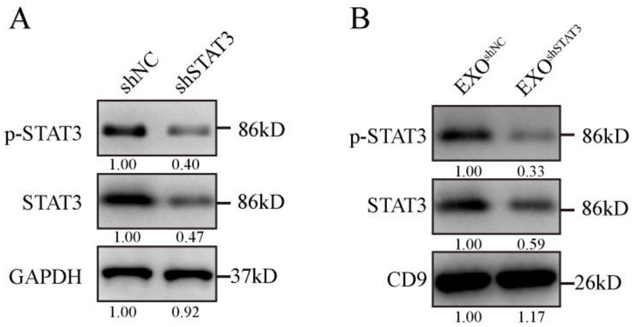

Supplementary Figure 6. A Western blot analysis of the efficiency of shRNA for STAT3 in HCT8 cells. **B** Western blot analysis of p-STAT3 and STAT3 levels in exosomes.

Supplemental Tables

Table S1 Correlation between Cyr61 levels and clinicopathologic characteristics of CRC patients

Characteristics	Frequency	Cyr61 expression level		
		Low	High	*p*-value[a]
Gender				0.984
Female	118	10	108	
Male	246	21	225	
Age				0.237
≤ 59	166	11	155	
> 59	198	20	178	
T stage				< 0.001
T1/T2	96	26	70	
T3/T4	268	5	263	
N stage				< 0.001
N0	192	28	164	
N1/N2	172	3	169	
M stage				0.034
M0	271	28	243	
M1	93	3	90	
TNM Stage				< 0.001
I+II	179	28	151	
II+IV	185	3	182	

[a] Chi-square test

Table S2 The ROC curve assay of CEA, CA199, CA125and Cyr61

Variables	ACU	*p*-value[a]	95 % Confidence interval	
			Lower bound	Upper bound
CEA	0.667	< 0.001	0.611	0.723
CA199	0.522	0.516	0.460	0.584
CA125	0.600	0.003	0.540	0.661
Cyr61	0.933	< 0.001	0.906	0.960

[a]Null hypothesis: true area = 0.5

Table S3 List of surface membrane proteins of mass spectrometry results (score>35)

Num	Prot_acc	Prot_desc	Prot_score	Prot_mass (KD)	Coverage	Unique peptides	PSM
1	P06756	Integrin alpha-V	3401	117.048	39.1	41	162
2	P18084	Integrin beta-5	1811	91.303	36.5	26	82
3	Q02413	Desmoglein-1	98	114.702	3.7	3	3
4	Q9Y5I4	Protocadherin alpha-C2	57	110.010	4.7	6	12
5	O00522	Krev interaction trapped protein 1	49	84.979	2.9	2	8
6	Q9Y5X5	Neuropeptide FF receptor 2	46	60.858	4.4	4	7
7	Q8NGB9	Olfactory receptor 4F6	38	35.787	2.6	1	2
8	Q12934	Filensin	36	74.784	2.3	2	4
9	O95196	Chondroitin sulfate proteoglycan 5	36	60.720	2.3	1	5

Table S4 Oligonucleotide sequences

Name	Sequences(5’- 3’)
18S	F: CGGCTACCACATCCAAGGAA
	R: GCTGGAATTACCGCGGCT
Cyr61	F: CGCCTTGTGAAAGAAACCCG
	R: GGTTCGGGGGATTTCTTGGT
CCN2	F: GTTTGGCCCAGACCCAACTA
	R: GGCTCTGCTTCTCTAGCCTG
CCN3	F: TGATGGTCATTGGGACCTGC
	R: GGTGCTCTGTAGGTGTGCTT
CCN4	F: GCGTGGAATGTGTTTGCTCA
	R: GCCTGTACAAGAAAAGCCACC
CCN5	F: CTGTGCCTCTGTAAGCAGGA
	R: AGAAGCGGTTCTGGTTGGAC
CCN6	F: CTCACTGCGAAGGCAGGTTA
	R: GTACCCTGCAGCAGAACTGT
shβ5	F:CCGGAAAGATGATGTGCCCCACATCGCATTGCTCGAGCAATG<br>CGATGTGGGGCACACATCTTTTTTG
	R:AATTCAAAAAAAAGATGATGTGCCCCACATCGCATTG<br>CTCGAGCAATGCGATGTGGGGCACATCATCT
shSTAT3	F:CCGGGGCCATCTTGAGCACTAAGCCCTCGAGGGCTTAGTGCT<br>CAAGATGGCC TTTTTG
	R:AATTCAAAAAGGCCATCTTGAGCACTAAGCCCTCGAGGGCTT<br>AGTGCTCAAGATGGCC
